# Supplementary material for: Hybridizing carbonate and ether at molecular scales for high-energy and high-safety lithium metal batteries
Source: Nat Commun. 2024 Apr 15;15:3217. doi: 10.1038/s41467-024-47448-5 (PMC11018806; doi:10.1038/s41467-024-47448-5)
Supplement: Supplementary file 1 — Supplementary information [file 41467_2024_47448_MOESM1_ESM.pdf]

Supplementary information for

Hybridizing Carbonate and Ether at Molecular Scales for High-energy and High-safety Lithium Metal Batteries

Jiawei Chen[1], Daoming Zhang[2], Lei Zhu[1], Mingzhu Liu[3], Tianle Zheng[4], Jie Xu[1],

Jun Li[2], Fei Wang[1], Yonggang Wang[1], Xiaoli Dong[1]* & Yongyao Xia[1]*

1. Department of Chemistry and Shanghai Key Laboratory of Molecular Catalysis and Innovative Materials, Institute of New Energy, iChEM (Collaborative Innovation Center of Chemistry for Energy Materials), Fudan University, Shanghai 200433, China.

2. Sinopec Shanghai Research Institute of Petrochemical Technology Co., Ltd., Shanghai 201208, China.

3. School of Chemistry, South China Normal University, Guangzhou 510006, China.

4. Department of Chemistry, College of Sciences, Shanghai University, Shanghai 200444, China.

* E-mail: xldong@fudan.edu.cn; yyxia@fudan.edu.cn

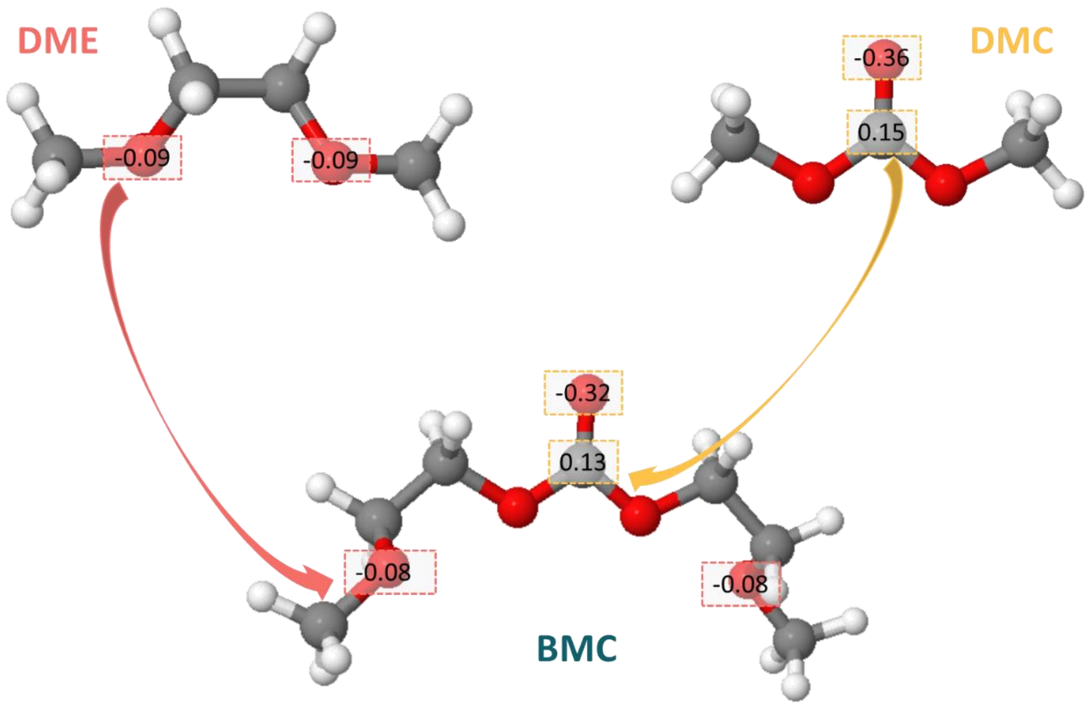

Supplementary Figure 1. Charge distribution (Mulliken type) of optimized DME, DMC and BMC. The gray, white and red balls stand for C, H and O atoms respectively.

Note: The ether oxygens in BMC show less negative charge than those in DME, while the carbonyl carbon and carbonyl oxygen in BMC respectively show less positive and less negative charge than those in DMC.

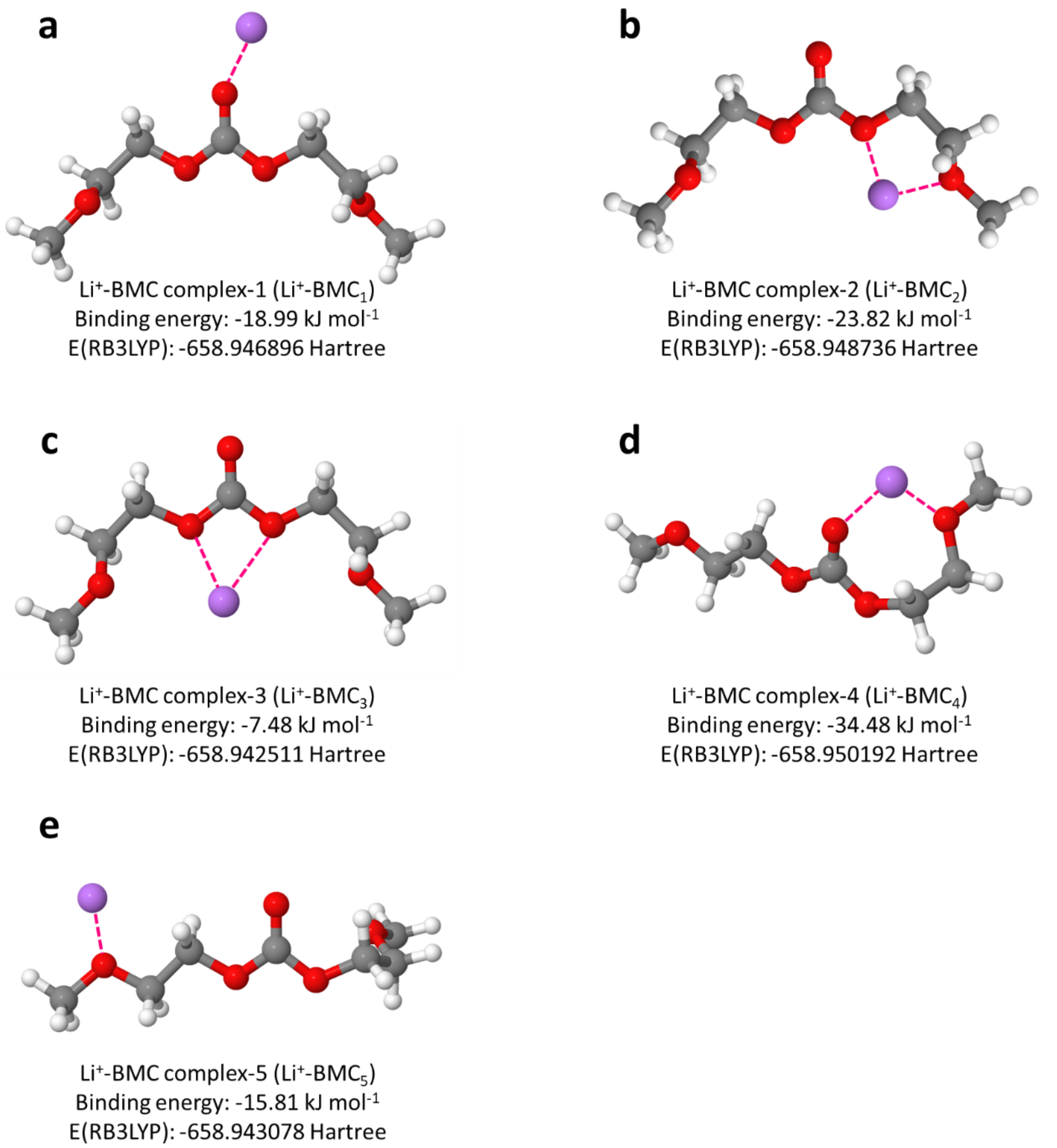

Supplementary Figure 2. DFT calculations for various Li^+-BMC complexes. Optimized structures, binding energy and electronic energy of (a) Li^+-BMC complex-1, (b) Li^+-BMC complex-2, (c) Li^+-BMC complex-3, (d) Li^+-BMC complex-4 and (e) Li^+-BMC complex-5. Purple, gray, white and red balls stand for Li^+, C, H and O, respectively.

Note: Multiple Li^+-BMC coordination configurations exist due to various potential coordinating sites (carbonyl and ether oxygen). Five configurations and their energy values are summarized in Supplementary Fig. 2: Li^+-BMC complex-1 (Li^+-BMC_1) which coordinates to Li^+ with carbonyl oxygen (binding energy: -18.99 kJ mol^{-1}), Li^+-BMC complex-2 (Li^+-BMC_2) whose five-membered ring chelation structure coordinates to Li^+ through two oxygen atoms (binding energy: -23.82 kJ mol^{-1}), Li^+-BMC complex-3 (Li^+-BMC_3) which coordinates to Li^+ with the single bond oxygens in the carbonate group (binding energy: -7.48 kJ mol^{-1}), Li^+-BMC complex-4 (Li^+-BMC_4) whose chelation structure coordinates to Li^+ through carbonyl and ether oxygens (binding energy: -34.48 kJ mol^{-1}), and Li^+-BMC complex-5 (Li^+-BMC_5) which coordinates to Li^+ with one ether oxygen atom (binding energy: -15.81

kJ mol^{-1}). Based on DFT calculations, Li^+-BMC complex-3 and Li^+-BMC complex-5 have relatively positive energy values, indicating their coordination configurations are less thermodynamically stable. Comparatively, Li^+-BMC complex-2 and Li^+-BMC complex-4 with strong chelation structures exhibit more negative energy values than other forms, suggesting their stability and potential as preferred coordination configurations. However, it is noteworthy that the DFT results have certain limitations as they solely consider scenarios where one Li^+ interacts with one solvent molecule, which is different from the coordination structure of Li^+ in real electrolyte involving complicated interactions and steric hindrance. This implies that the partially surrounded chelation structure formed from their two solvating sites in Li^+-BMC complex-2 and Li^+-BMC complex-4 would hinder more coordinated molecules to participate in the Li^+ primary solvation structure of real electrolyte. Considering these complex factors, MD simulations, as conducted below, can output a more compelling distribution of solvation configuration, where Li^+-BMC complex-1 represents the dominant coordination configuration where carbonyl oxygen solely coordinates to Li^+. This conclusion will be further confirmed by experimental results later, including the marginal solubility of $LiNO_3$ and Raman spectra.

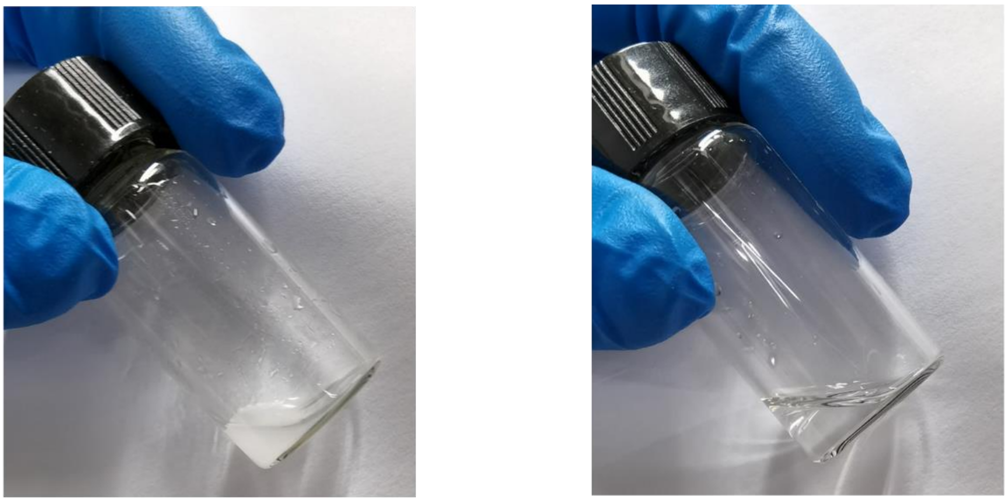

1.2m LiFSI in DMC + 0.75 wt.% $LiNO_3$ 1.2m LiFSI in BMC + 0.75 wt.% $LiNO_3$

Supplementary Figure 3. Optical images of $LiNO_3$-containing electrolytes.

Note: The dissolution of salt in a solvent is primarily determined by the competition between cation-anion interaction in solid salt and ion-solvent interaction in solution[1]. Therefore, it is generally considered that a stronger interaction between Li^+ and solvent would facilitate the dissolution of salt. $LiNO_3$ is found hardly dissolved in DMC-based electrolyte (binding energy of Li^+-DMC: -19.24 kJ mol^{-1}), which is consistent with its widely-reported low solubility in carbonate electrolytes (< 10^{-5} g mL^{-1}, corresponding to < 0.1 wt %)[2,3]. The commonly-used DME-based electrolyte (binding energy of Li^+-DME: -43.06 kJ mol^{-1}) exhibited a much higher solubility of $LiNO_3$ (typically 5 wt %)[4]. As for BMC, the binding energies of Li^+-BMC complex-1, Li^+-BMC complex-3 and Li^+-BMC complex-5 are even weaker than that of Li^+-DMC, whereas the binding energies of other two chelation configurations (Li^+-BMC complex-2: -23.82 kJ mol^{-1} and Li^+-BMC complex-4: -34.48 kJ mol^{-1}) fall in between Li^+-DMC and Li^+-DME. Consequently, the capability of BMC to dissolve $LiNO_3$ might be attributed to the Li^+-BMC complex-2 and Li^+-BMC complex-4 chelation configurations with double coordinating sites. However, the upper limit of the $LiNO_3$-solubility in BMC is approximately 0.75 wt %, which is still much lower than that in DME. Such phenomenon implies that Li^+-BMC complex-2 and Li^+-BMC complex-4 might not be the dominant coordination configurations in BMC-based electrolyte, which will be further verified in the MD simulation and Raman spectra later.

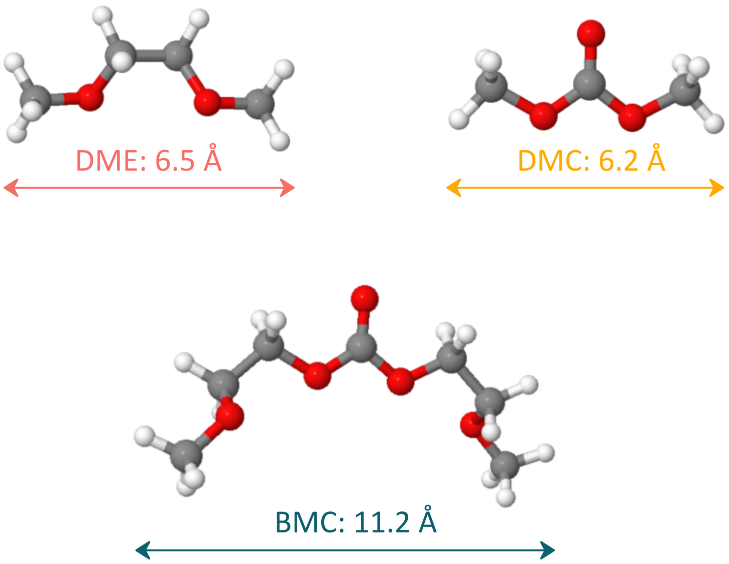

Supplementary Figure 4. Molecular dimensions of different solvents obtained from DFT calculations.

Note: It can be detected that introducing CH_3OCH_2- moieties at both ends of DMC to hybridize ether and carbonate within one molecule significantly extended the length of BMC to 11.2 Å, much longer than those of DME (6.5 Å) and DMC (6.2 Å). The large molecular size of BMC introduces significant steric hindrance, presenting challenges in effectively accommodating multiple BMC molecules within the Li^+ primary solvation sheaths. Consequently, this increased steric effect could control and weaken the solvation capability of BMC and facilitates the ingress of more FSI^- into the inner solvation shell.

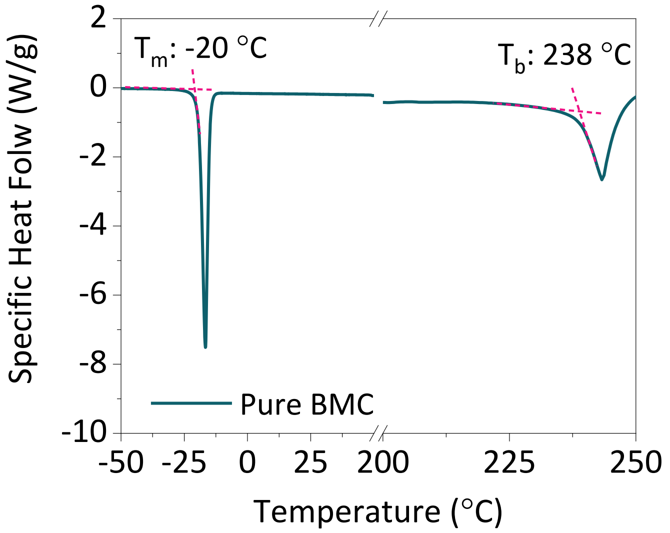

Supplementary Figure 5. DSC curve of pure BMC solvent at a heating rate of 5 °C min^{-1} from -50 to 250 °C. (T_m: melting point; T_b: boiling point)

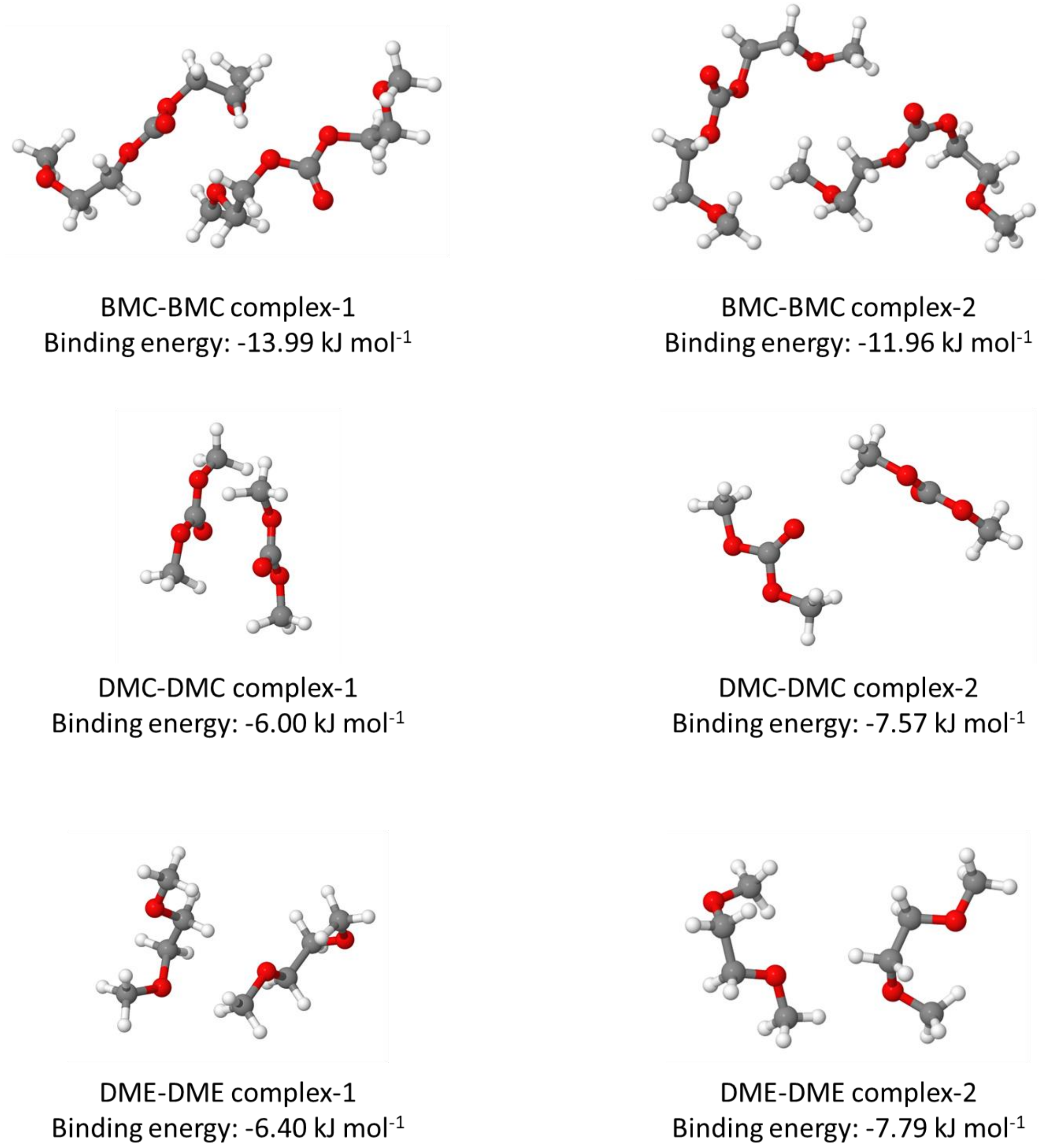

Supplementary Figure 6. DFT calculations for various solvent-solvent complexes. Optimized structures and binding energy of BMC-BMC, DMC-DMC and DME-DME complexes that are depicted by the ball-and-stick model. The gray, white, and red balls represent carbon (C), hydrogen (H), and oxygen (O) atoms, respectively. All complexes were directly extracted from the corresponding molecular dynamics (MD) simulation results and optimized using the Gaussian 09 software package at the 3LYP/6-311++G(d) level.

Note: According to the DFT calculation results shown in Supplementary Fig. 6, the binding energy between two BMC molecules (-13.99 kJ mol^{-1}; -11.96 kJ mol^{-1}) is almost twice than that of two DMC molecules (-6.00 kJ mol^{-1}; -7.57 kJ mol^{-1}) or two DME molecules (-6.40 kJ mol^{-1}; -7.79 kJ mol^{-1}), indicating that the van der Waals force between BMC solvent is significantly enhanced by extending the molecular chain. The stronger intermolecular force of BMC requires a higher vaporization enthalpy, thereby increasing the boiling point (238 °C) of BMC. Moreover, the strong intermolecular force of BMC decreases the saturated vapor pressure, increasing the flash point to 117 °C. Such high flash point

equips the electrolyte based on BMC solvent with advantages in view of safety compared to DMC and DME.

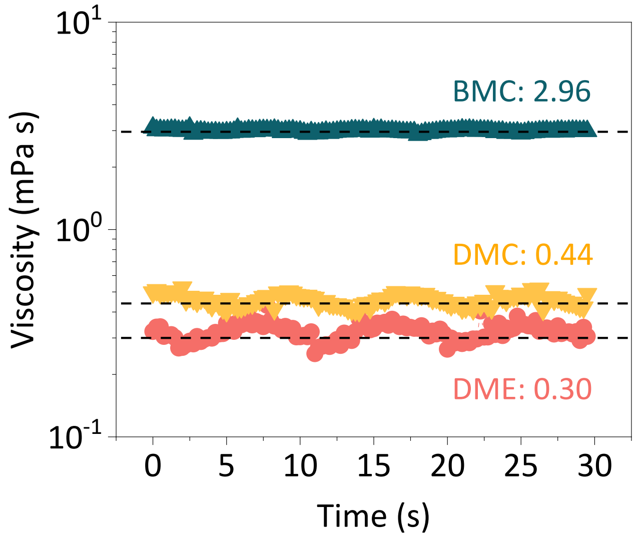

Supplementary Figure 7. Viscosity tests for different solvents.

Note: The viscosity values are 0.3 mPa s for DME, 0.44 mPa s for DMC, and 2.96 mPa for BMC respectively.

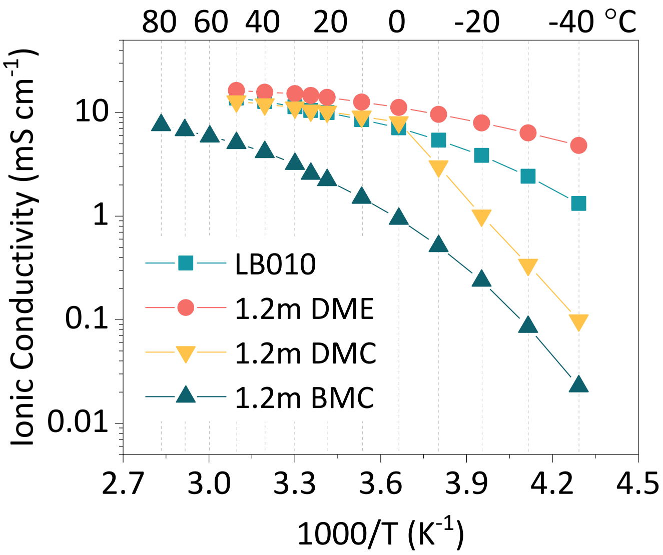

Supplementary Figure 8. Ionic conductivity of different electrolytes at various temperatures.

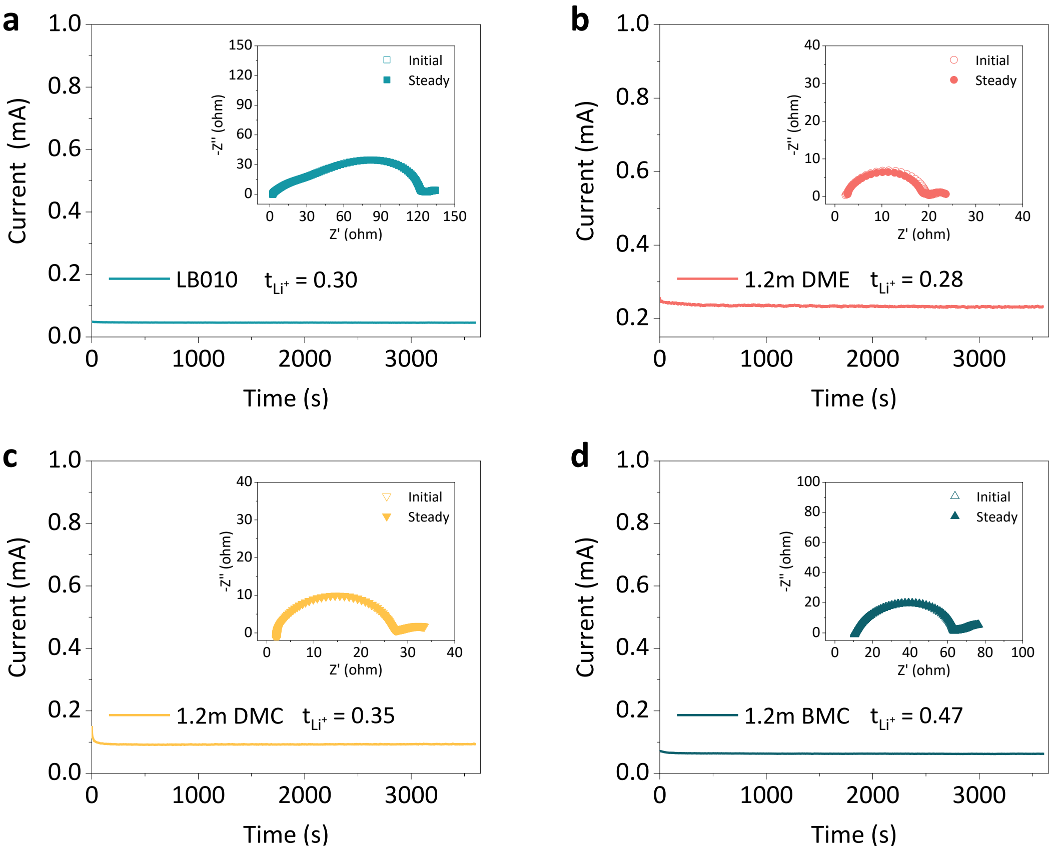

Supplementary Figure 9. Li^+ transfer number measurements of various electrolytes. Chronoamperometry (CA, under a voltage of 10 mV) curves of Li||Li symmetric cells operated in (a) LB010, (b) 1.2m DME, (c) 1.2m DMC and (d) 1.2m BMC, together with the corresponding EIS profiles (see insets) before and after CA tests and calculated Li^+ transfer numbers.

Note: The detailed calculation procedure of the Li^+ transfer number can be referred to the previous work[5].

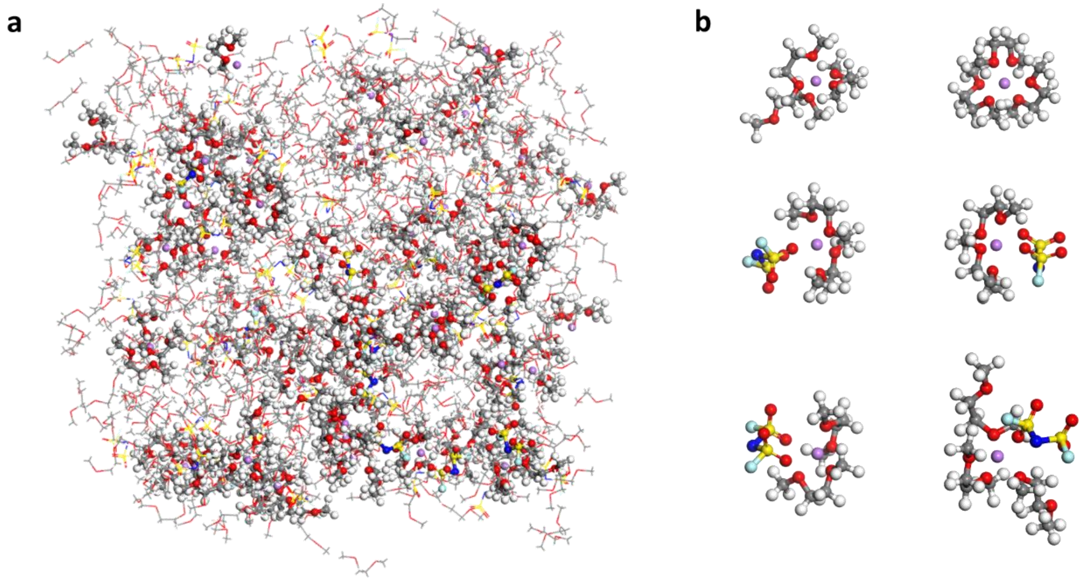

Supplementary Figure 10. MD simulation results for 1.2m DME. (a) MD simulated electrolyte structure for 1.2m DME. Li^+ and coordinated molecules (within 2.5 Å of Li^+) are depicted by the ball-and-stick model. Purple balls stand for Li ions, while gray, white, red, blue, yellow and cyan balls stand for C, H, O, N, S and F atoms respectively. (b) Some corresponding representative Li^+ solvation structures extracted from the MD simulation.

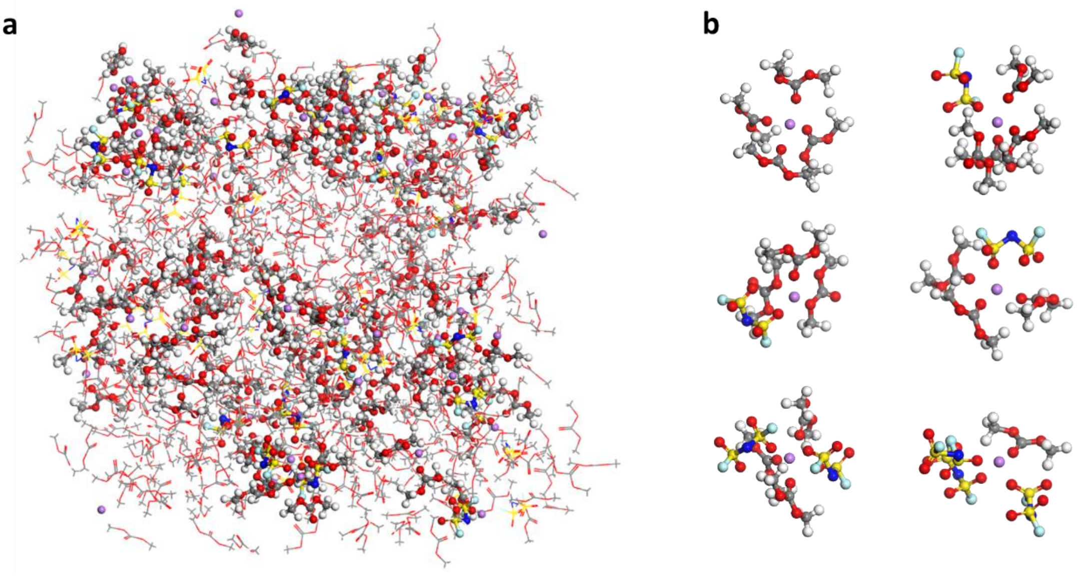

Supplementary Figure 11. MD simulation results for 1.2m DMC. (a) MD simulated electrolyte structure for 1.2m DMC. Li^+ and coordinated molecules (within 2.5 Å of Li^+) are depicted by the ball-and-stick model. Purple balls stand for Li ions, while gray, white, red, blue, yellow and cyan balls stand for C, H, O, N, S and F atoms respectively. (b) Some corresponding representative Li^+ solvation structures extracted from the MD simulation.

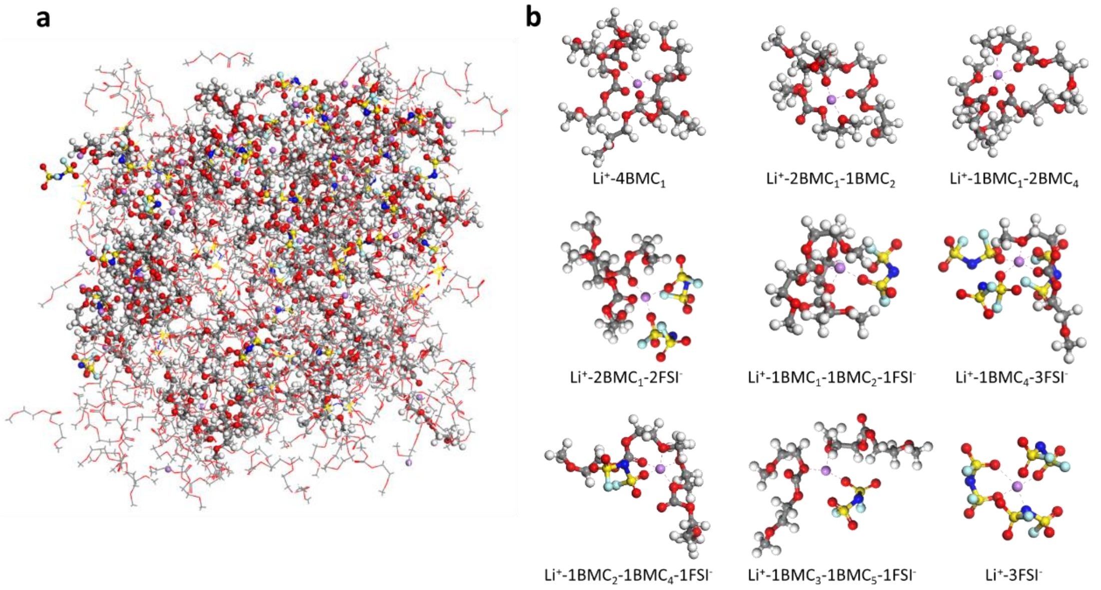

Supplementary Figure 12. MD simulation results for 1.2m BMC. (a) MD simulated electrolyte structure for 1.2m BMC. Li^+ and coordinated molecules (within 2.5 Å of Li^+) are depicted by the ball-and-stick model. Purple balls stand for Li ions, while gray, white, red, blue, yellow and cyan balls stand for C, H, O, N, S and F atoms respectively. (b) Some corresponding representative Li^+ solvation structures extracted from the MD simulation. (Li^+-BMC_1, Li^+-BMC_2, Li^+-BMC_3, Li^+-BMC_4 and Li^+-BMC_5 can correspond to Li^+-BMC complex-1, Li^+-BMC complex-2, Li^+-BMC complex-3, Li^+-BMC complex-4 and Li^+-BMC complex-5 shown in DFT results, respectively.)

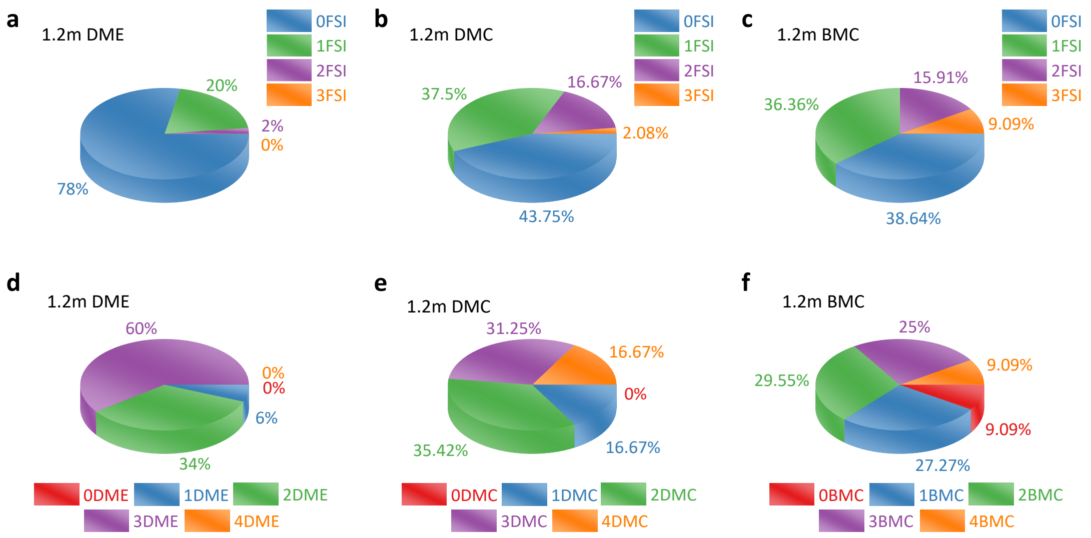

Supplementary Figure 13. The distributions of various Li^+ coordination environments for 1.2m DME, 1.2m DMC and 1.2m BMC. The Li^+ coordination environments are categorized according to the number of (a-c) FSI^- and (d-f) solvent molecules in Li^+ PSSs.

Note: The average numbers of FSI^- participating in Li^+ PSSs of 1.2m DME, 1.2m DMC and 1.2m BMC are 0.24, 0.77 and 0.95, respectively; whereas the average number of solvent molecules participating in Li^+ PSSs for these electrolytes are found to be 2.54, 2.48, and 1.98 correspondingly.

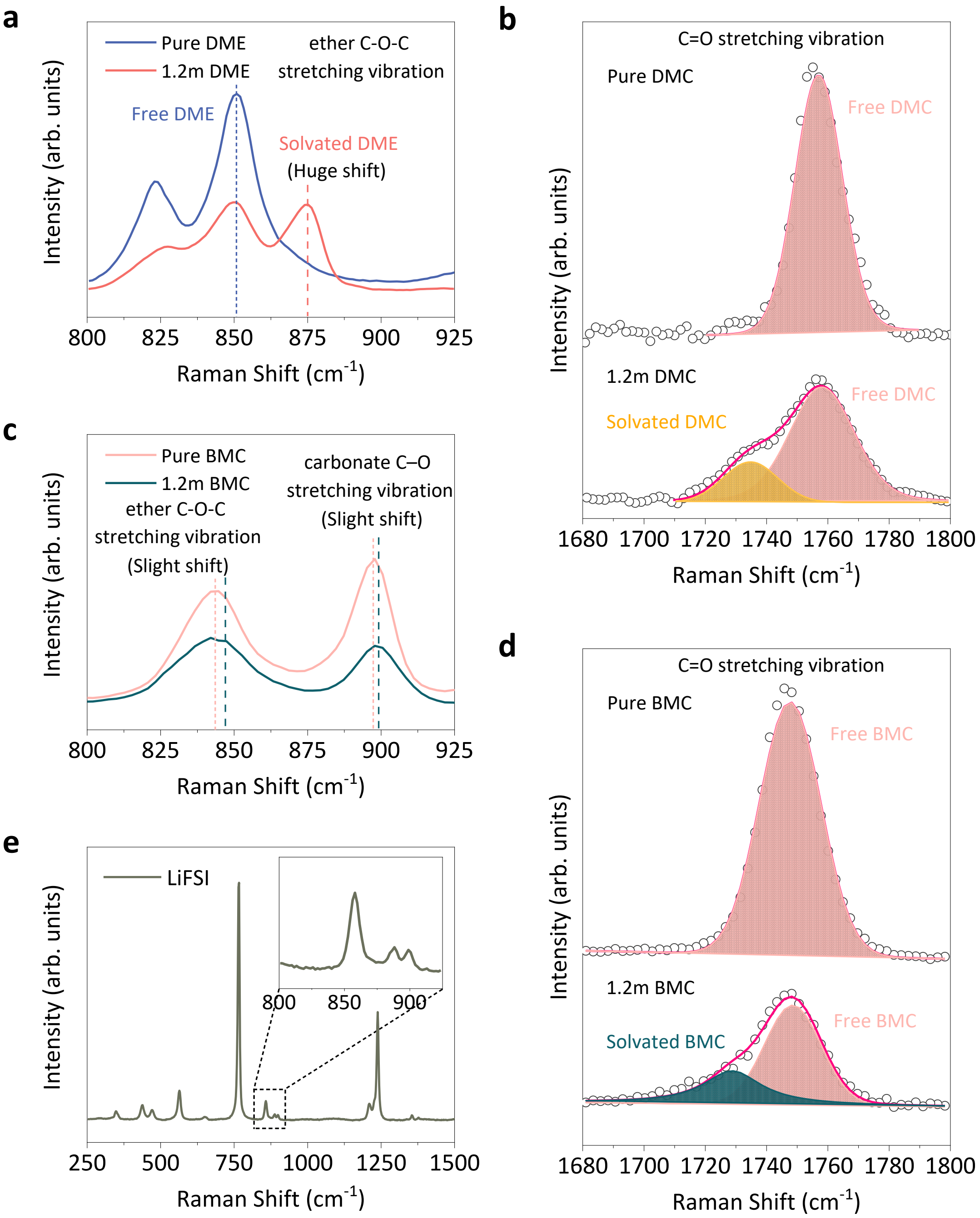

Supplementary Figure 14. Raman spectra of various electrolytes. (a) Raman spectra of 1.2m DME compared with pure DME in 800-925 cm^{-1} (ether C-O-C stretching vibration). (b) Fitted Raman spectra of 1.2m DMC compared with pure DMC in 1680-1800 cm^{-1} (carbonate C=O stretching vibration). Raman spectra of 1.2m BMC compared with pure BMC in (c) 800-925 cm^{-1} (ether C-O-C and carbonate C-O stretching vibrations) and (d) 1680-1800 cm^{-1} (carbonate C=O stretching vibration). (e) Raman spectra of LiFSI.

Note: As shown in Supplementary Fig. 14a, the introduction of LiFSI in pure DME leads to a notable blue shift in C-O-C stretching vibrations (851 cm^{-1}), resulting in a new peak (875 cm^{-1}) corresponding to solvated DME. This well indicates the coordination of some DME molecules with Li^+ through their sole solvation site, the ether oxygen. Supplementary Fig. 14b presents the Raman spectra of pure DMC

and 1.2m DMC. The carbonate C=O stretching vibration peak of free (uncoordinated) DMC locates at 1757 cm^{-1}. Upon adding LiFSI to DMC, a new peak emerges at 1735 cm^{-1}, corresponding to the coordination of DMC to Li^{+} with its carbonyl oxygen. In contrast, BMC exhibits slight shifts in carbonate C-O and ether C-O-C vibrations upon the introduction of LiFSI (Supplementary Fig. 14c), which is quite different from that observed in 1.2m DME. In contrast, a new peak locating at 1729 cm^{-1} arises near the carbonate C=O stretching vibration (1748 cm^{-1}, Supplementary Fig. 14d), which is similar to 1.2m DMC and should correspond to the solvated BMC. Such results can effectively demonstrate that carbonyl oxygen is the main solvating site of BMC, and Li^{+}-BMC complex-1 is the most prevalent coordination configuration. Moreover, it can be detected that regardless of the solvent systems, there will always be a certain reduction in peak intensities for solvents after incorporating LiFSI. Noteworthily, the relative change between carbonate C-O and ether C-O-C stretching vibrations shown in Supplementary Fig. 14c might be interfered by the antisymmetric stretching vibration of S-N-S from LiFSI at around 850 cm^{-1}, which slightly overlaps with that of ether C-O-C stretching vibrations (Supplementary Fig. 14e)[6].

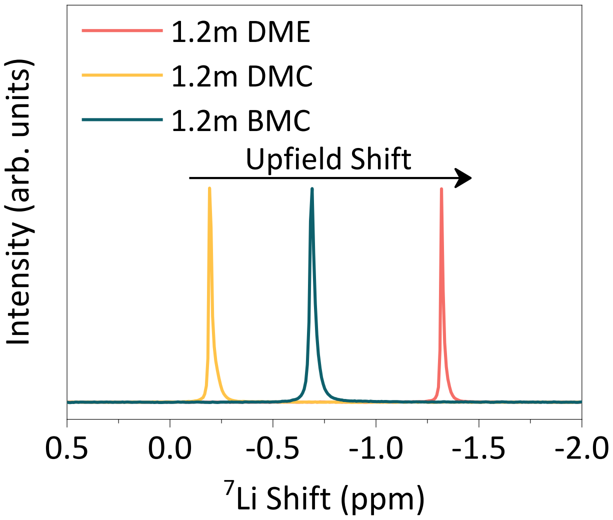

Supplementary Figure 15. ^{7}Li NMR spectra of various electrolytes. The chemical shift is externally referenced to 1M LiCl in H_2O (0.00 ppm).

Note: ^{7}Li NMR spectra are sensitive to the Li^+ coordination environments. In 1.2m BMC, an upfield (more negative) shift was found while compared with 1.2m DMC, suggesting an increased Li^+-FSI^- ion pairing. However, 1.2m DME is even more upfield shifted than 1.2m BMC, which may be due to the super solvent binding between Li^+ and DME rather than the increased ion pairing of Li^+ with FSI^-[7].

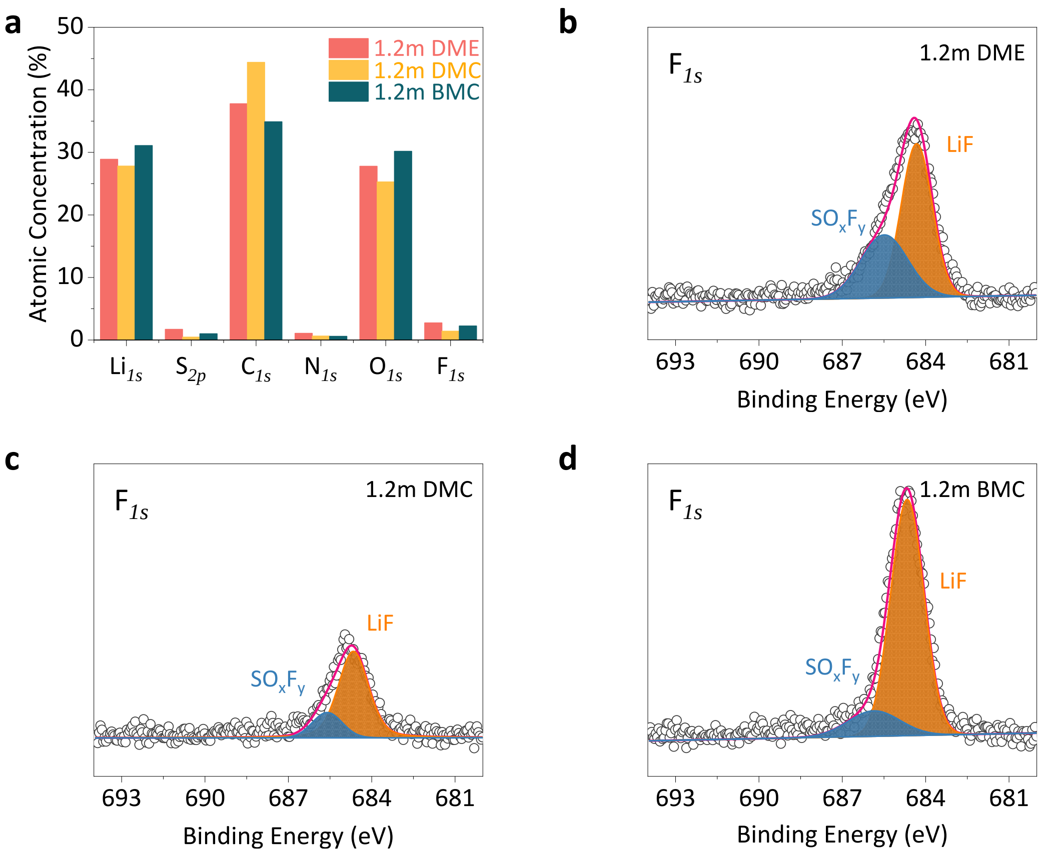

Supplementary Figure 16. XPS results of SEI formed in different electrolytes. (a) Atomic concentration of detected elements on SEI formed in different electrolytes, and corresponding (b-d) F_{1s} XPS patterns.

Note: The XPS results clearly show that the SEI formed in 1.2m BMC contains the most LiF species, while the one in 1.2m DME and 1.2m DMC contains fewer and fewest, respectively. Although the Li^+ PSSs of 1.2m DMC contains a considerable amount of FSI^-, the reductive stability of DMC solvent itself is so poor to the point where the reduction of DMC plays a dominant role in the SEI-forming process (see the towering carbon content within 1.2m DMC-derived SEI) and hinders the generations of LiF species.

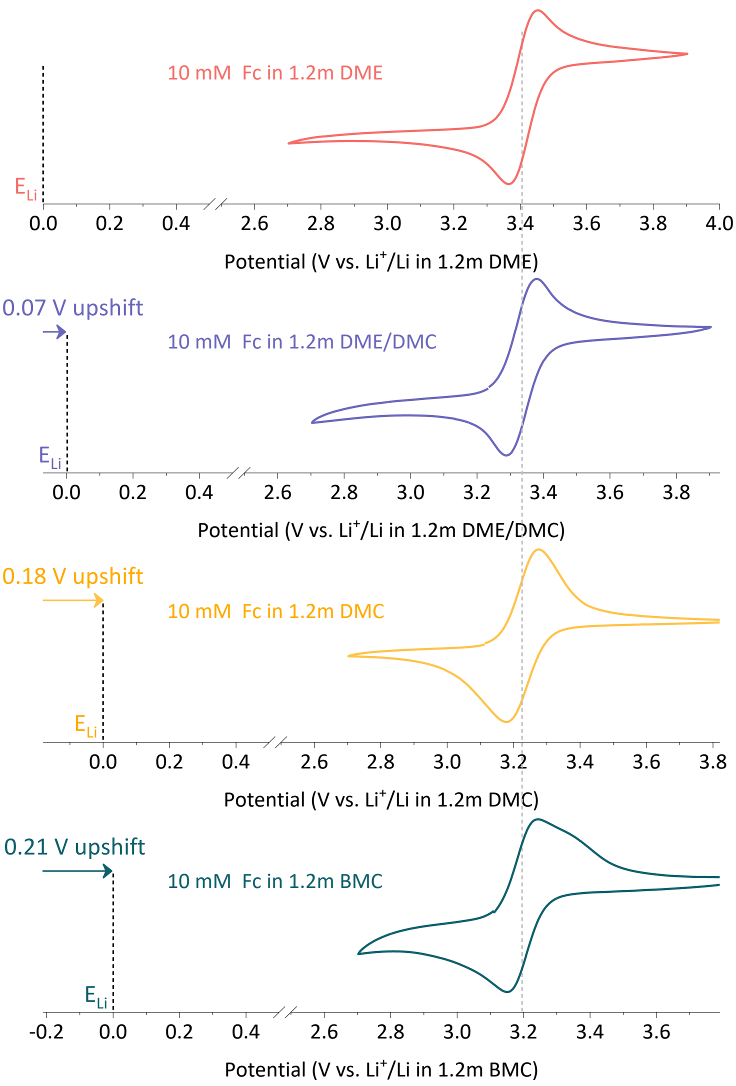

Supplementary Figure 17. CV curves of 10 mM Fc in various electrolytes with Pt as working electrode at 5 mV s^{-1}. The profiles are aligned according to the redox of Fc^+/Fc to make it easier to see the upshifts of E_{Li} in 1.2m DME/DMC, 1.2m DMC and 1.2m BMC. The E_{Li} in 1.2m DME is set as reference.

Note: After aligning the CV curves based on the redox potential of Fc^+/Fc, a more visually intuitive comparison of E_{Li} in different electrolytes can be observed. Specifically, 1.2m DME/DMC, 1.2m DMC and 1.2m BMC show an upshift of E_{Li} by 0.07 V, 0.18 V and 0.21 V, respectively, compared to that in 1.2m DME. Considering that the E_{Li} value is influenced by the degree of Li^+-FSI^- ion pairing, it can be inferred that the solvation structure of the electrolyte is lightly affected by mere physical blending of ether and carbonate (1.2m DME/DMC). By contrast, the intramolecularly hybridized BMC enables significantly enhanced incorporation of FSI^- into the Li^+ primary solvation sheaths (PSSs) for the formation of Li^+-FSI^- ion pairing due to its relatively weak solvating power and steric effect. Obviously, the E_{Li} tested in 1.2m BMC experiences the greatest upshift, effectively shortening the gap between redox of Li^+/Li and potential window of electrolyte, considerably diminishing the undesired electrolyte decomposition and improving the Li anode CE. These results underscore the utmost importance of thoroughly altering physicochemical properties of solvents at the molecular level.

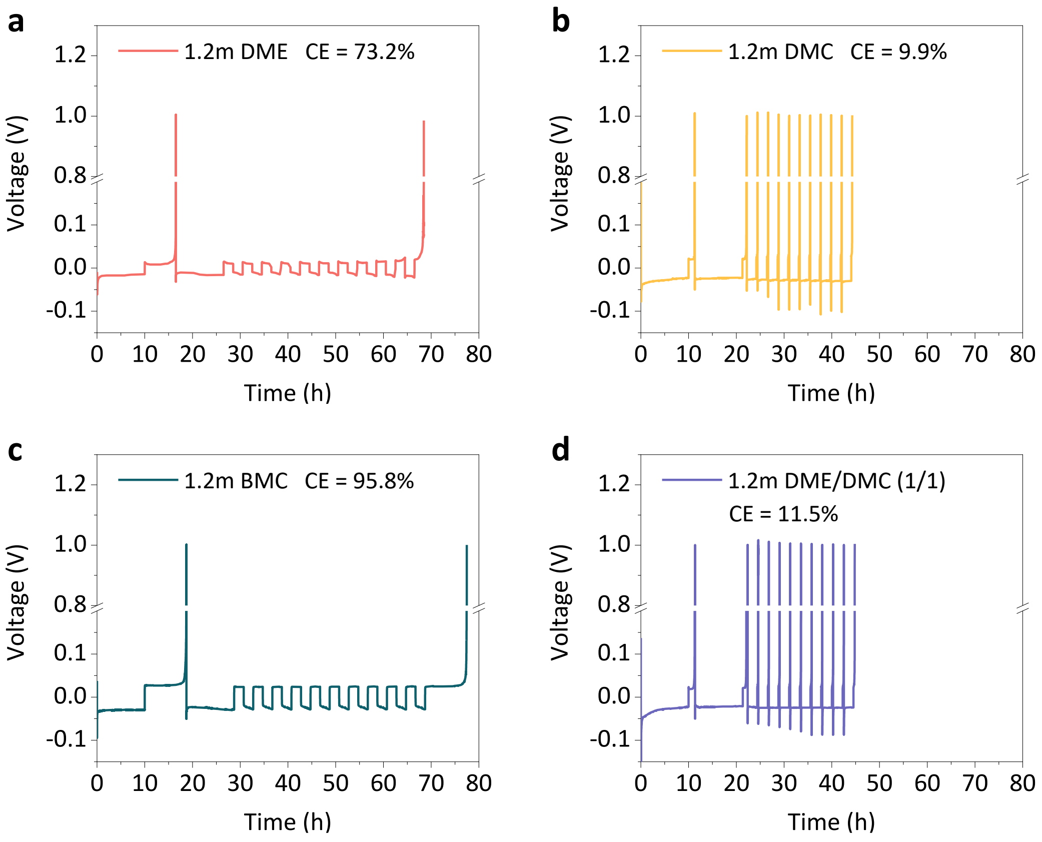

Supplementary Figure 18. Voltage profiles of 10 cycles-Li plating/stripping CE tests performed at 0.5 mA cm^{-2} and 1 mAh cm^{-2} in various electrolytes.

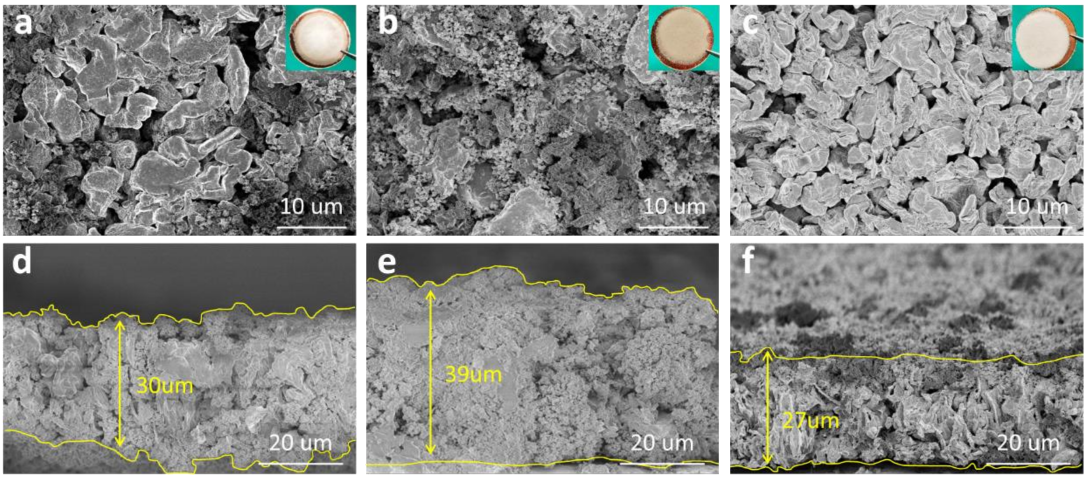

Supplementary Figure 19. Morphology of Li electrodeposit formed in various electrolytes**.** SEM images for the (a-c) surface and (d-f) cross-section of Li electrodeposit after the first plating on the Cu foil at 0.5 mA cm^{-2} and 3 mAh cm^{-2} using (a, d) 1.2m DME, (b, e) 1.2m DMC and (c, f) 1.2m BMC. The insets in a-c are optical images of Li electrodeposit on the Cu foil.

Note: As can be seen from the respective optical images, deposited Li in both 1.2m DME and 1.2m BMC stay shiny, whereas severe side reactions tarnish the Li plating in 1.2m DMC. Porous structure and particulate 'dead Li' with large specific surface area can be seen everywhere for SEM images of Li electrodeposit in 1.2m DMC. The situation is somewhat improved in the 1.2m DME, and the Li particles are bulkier. As for the Li electrodeposit in 1.2m BMC, it presents the most uniform, dense and flat morphology.

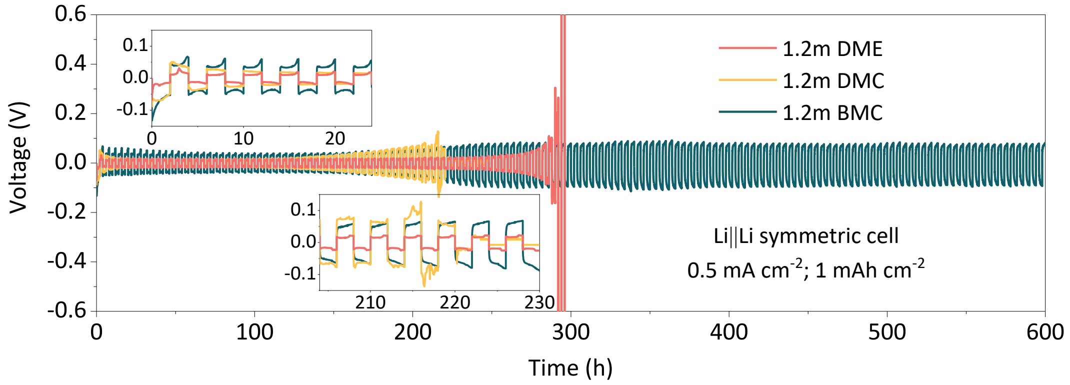

Supplementary Figure 20. Cycling performance of Li||Li symmetric cells operated in 1.2m DME, 1.2m DMC and 1.2m BMC.

Note: During cycling at 0.5 mA cm^{-2} and 1 mAh cm^{-2}, the Li||Li symmetric cell operated in the 1.2m DMC experiences a short circuit after 220 h due to the dendrite growth. For the cell operated in 1.2m DME, a significant increase in polarization is observed at 280 h, indicating the accumulation of resistive ‘dead Li’ and electrolyte depletion. However, despite initially exhibiting higher voltage polarization due to low ionic conductivity, the cell cycled in 1.2m BMC demonstrates superior stability by not significantly increasing its polarization even after 600 h.

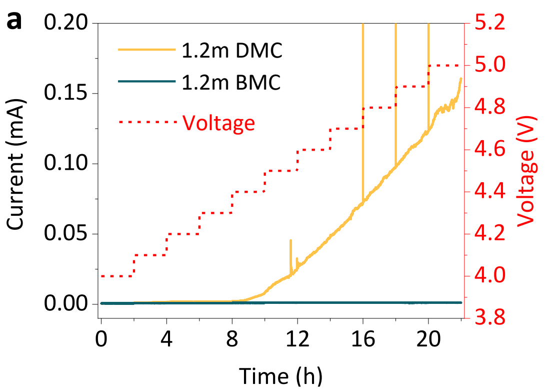

Supplementary Figure 21. CA tests of Al foils. (a) CA curves and (b) their zoomed-in plots of Al foils performed in 1.2m DMC and 1.2m BMC.

Note: The higher residual current indicates the more serious Al corrosion. In 1.2m BMC, during gradually raising the voltage from 4.0 to 5.0 V, the increase on the current is negligible, a clear indication for remarkable anti-corrosion to Al of the BMC-based electrolyte.

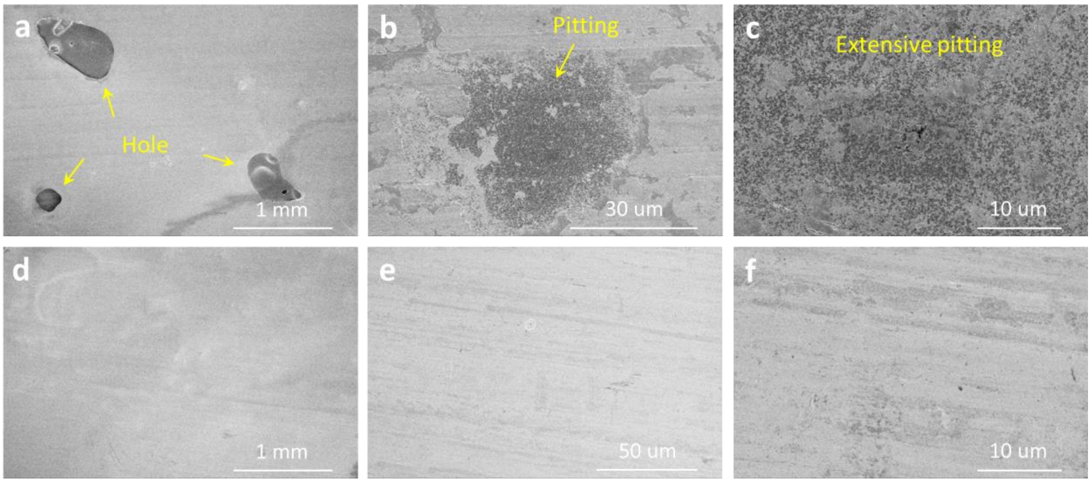

Supplementary Figure 22. Al corrosions in different electrolytes. SEM images of Al foils after the CA tests conducted in (a-c) 1.2m DMC and (d-f) 1.2m BMC.

Note: After the CA tests in Supplementary Fig. 21, significant holes and extensive pitting are shown on the Al foil measured in 1.2m DMC, revealing severe Al corrosions. On the contrary, smooth surface with no corrosions is found on the Al foil after tested in 1.2m BMC.

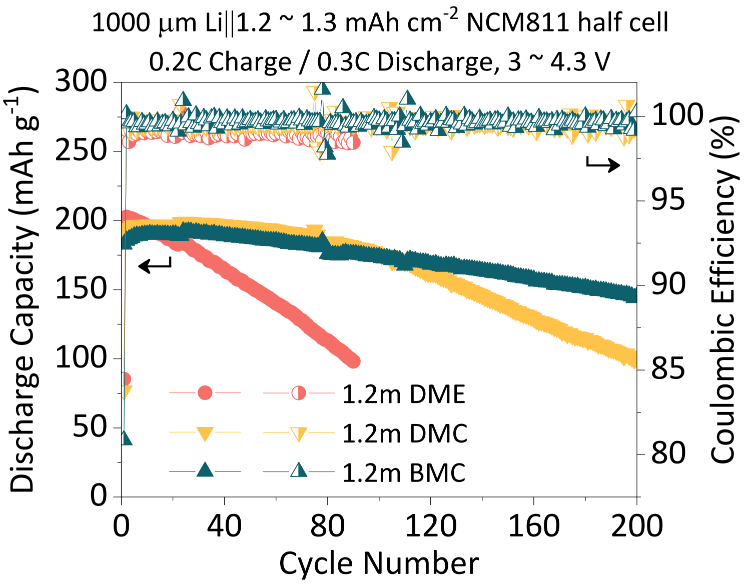

Supplementary Figure 23. Cycling performance of Li||NCM811 half-cells operated in various electrolytes.

Note: The Li||NCM811 half-cell operated in 1.2m BMC (average CE: 99.7%) provides better cycling performance than those operated in 1.2m DME (average CE: 99.0%) and 1.2m DMC (average CE: 99.6%).

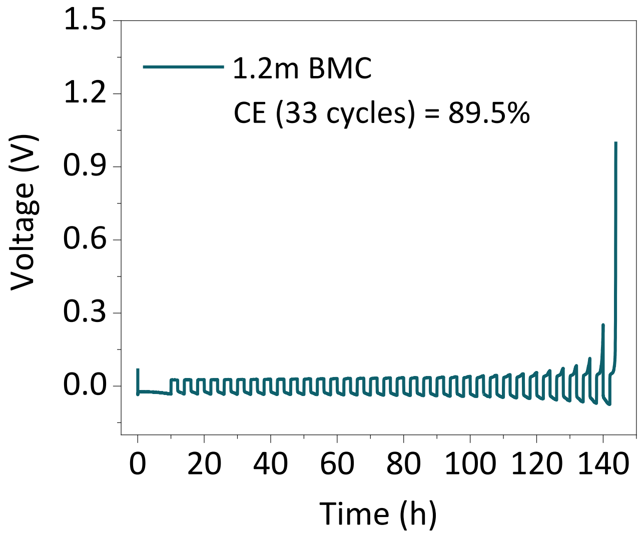

Supplementary Figure 24. Long-term Li plating/stripping CE test performed at 0.5 mA cm^{-2} and 1 mAh cm^{-2} in 1.2m BMC.

Note: After 33 cycles of Li plating/stripping in 1.2m BMC, all of the Li reservoir is depleted, corresponding to a Li anode CE of 89.5%.

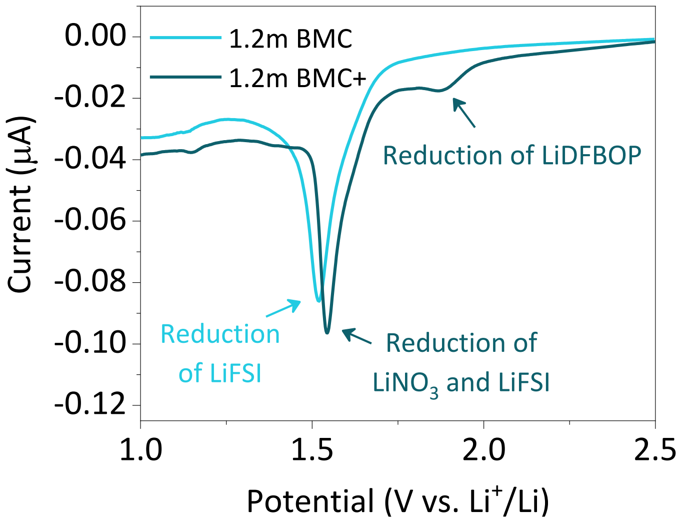

Supplementary Figure 25. LSV measurement on the Cu foil at a scan rate of 1 mV s^{-1} in 1.2m BMC with and without additives.

Note: LSV measurement was conducted on the Cu foil to confirm the involvement of additives in constructing SEI in 1.2m BMC+. It can be observed that, after the introduction of additives, a new reduction peak emerges at an earlier potential of 1.9 V vs. Li^+/Li, which can be attributed to the reduction of LiDFBOP[8]. Meanwhile, the slight shift to the right for the reduction peak around 1.5 V vs. Li^+/Li should be ascribed to the participation of $LiNO_3$[9].

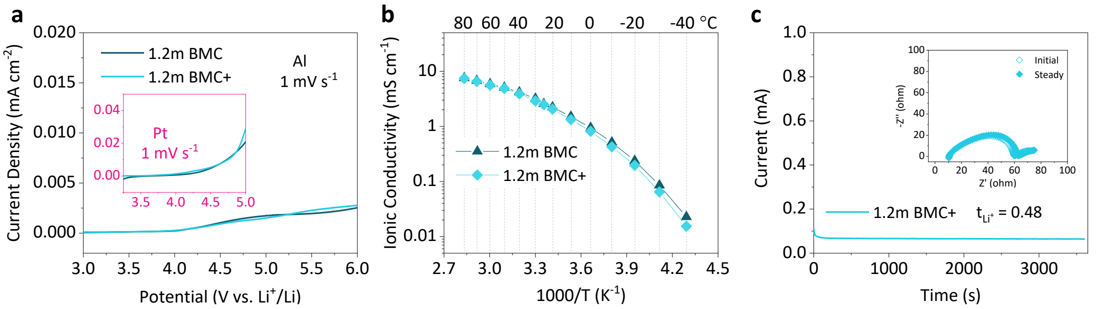

Supplementary Figure 26. Some physicochemical tests on additive-containing 1.2m BMC+ electrolyte. (a) Oxidative stability of 1.2m BMC and 1.2m BMC+ electrolytes measured with Al working electrodes at 1 mV s^{-1} (The inset is results obtained with Pt working electrodes). (b) Ionic conductivity of 1.2m BMC+ at various temperatures compared with that of 1.2m BMC. (c) Chronoamperometry (CA, under a voltage of 10 mV) curves of Li||Li symmetric cells operated in 1.2m BMC+, together with the corresponding EIS profiles (see insets) before and after the CA test and the calculated Li^+ transfer number.

Note: As demonstrated in Supplementary Fig. 26a, regardless of whether Al or Pt is used as the working electrode, the LSV results imply that the addition of $LiNO_3$ and LiDFBOP additives has a minor impact on the oxidative stability of the BMC-based electrolyte. Moreover, as presented in Supplementary Figs. 26b and 26c, it can be found that the effect is also negligible on both the ionic conductivity (25°C: 2.48 mS cm^{-1} (1.2m BMC+) vs. 2.56 mS cm^{-1} (1.2m BMC)) and Li^+ transfer number (0.48 (1.2m BMC+) vs. 0.47 (1.2m BMC)) of electrolyte upon the introduction of additives.

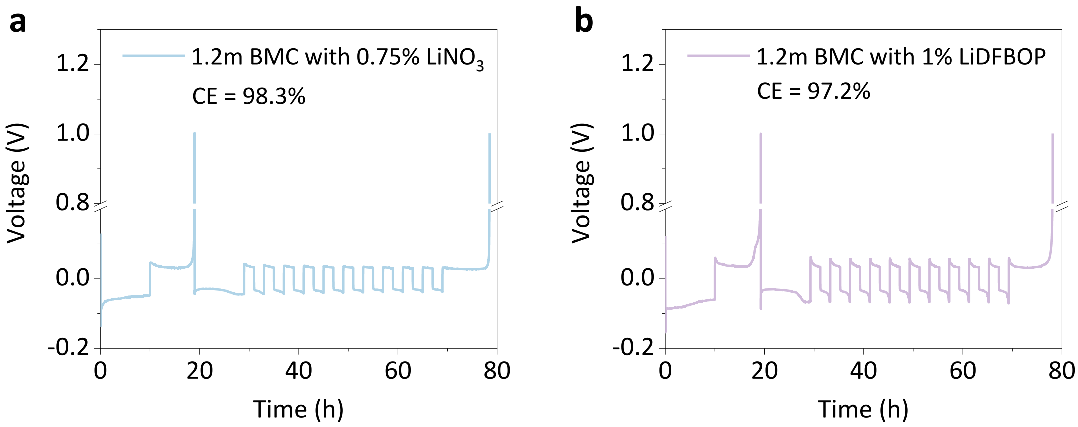

Supplementary Figure 27. 10 cycles Li plating/stripping CE test performed at 0.5 mA cm^{-2} and 1 mAh cm^{-2} in BMC-based electrolytes with different additives: (a) $LiNO_3$ and (b) LiDFBOP.

Note: When solely incorporating $LiNO_3$ or LiDFBOP into the BMC-based electrolyte, the achieved Li plating/stripping CE is 98.3% or 97.2%, respectively, which is lower compared to the value attained through the synergistic effect of both $LiNO_3$ and LiDFBOP additives. It seems that the presence of $LiNO_3$-derived N-containing species (LiN_xO_y and Li_3N) and LiDFBOP-derived P-containing species (P-O and P-F) appears to be crucial for the formation of the optimal SEI, with neither being dispensable. Therefore, 1.2m BMC containing both $LiNO_3$ and LiDFBOP (1.2m BMC+) is considered as the optimal electrolyte for further research.

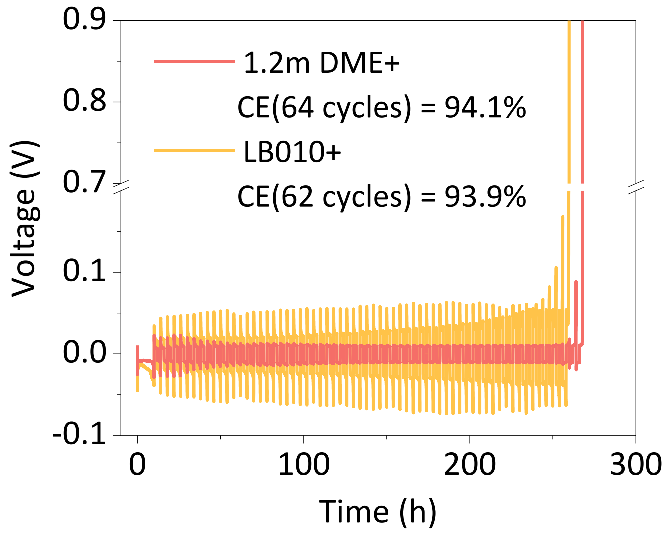

Supplementary Figure 28. Long-term Li plating/stripping CE test performed at 0.5 mA cm^{-2} and 1 mAh cm^{-2} in 1.2m DME+ and LB010+.

Note: The entire Li reservoir is fully exhausted after 64 and 62 cycles of Li plating/stripping in 1.2m DME+ and LB010+, respectively, indicating a Li anode CE of 94.1% and 93.9%.

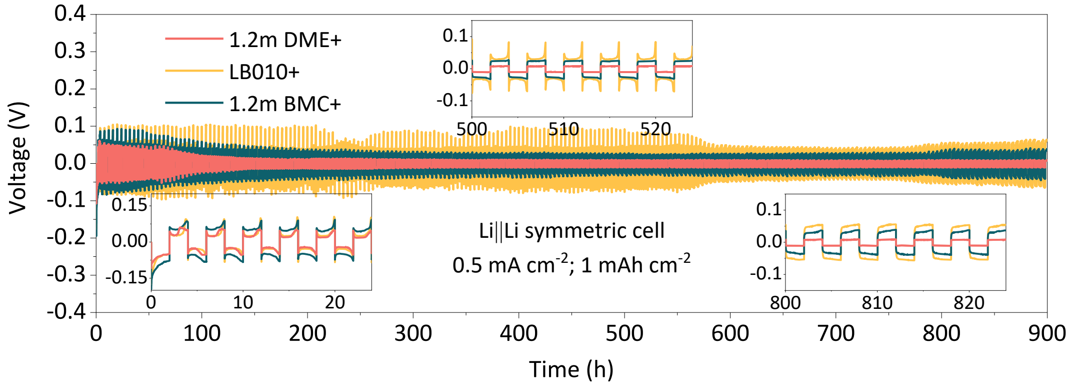

Supplementary Figure 29. Cycling performance of Li||Li symmetric cells operated in 1.2m DME+, LB010+ and 1.2m BMC+.

Note: During cycling at 0.5 mA cm^{-2} and 1 mAh cm^{-2}, the Li||Li symmetric cells operated in 1.2m BMC+ and 1.2m DME+ exhibit exceptional cycling stability over a period of 900 h, with no observed increase in voltage polarization. However, the cell using LB010+ shows a gradual and slight increase in voltage polarization and surpasses that of the cell using 1.2m BMC+, although it does not fail within 900 h.

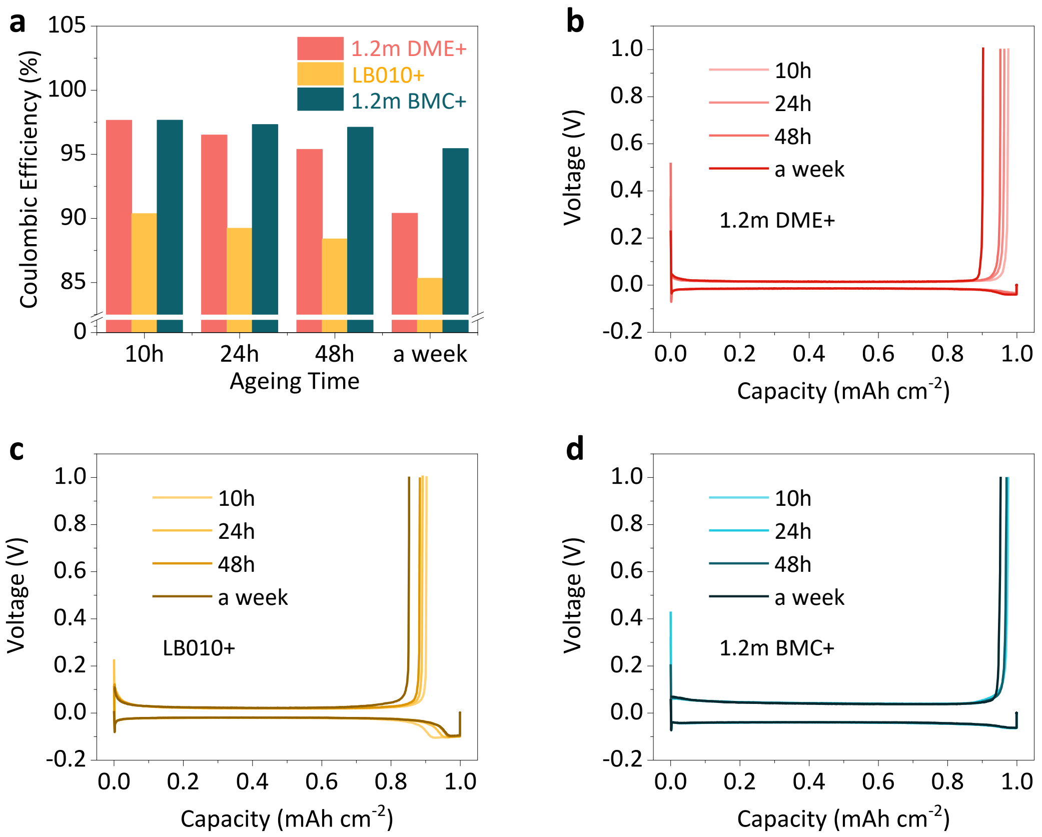

Supplementary Figure 30. Calendar ageing (10 h, 24 h, 48 h and a week) tests for Li||Cu half cells conducted at 0.5 mA cm^{-2} and 1 mAh cm^{-2} in various electrolytes: (a) results and (b-d) corresponding Li plating/stripping profiles during testing.

Note: It can be found that the optimized 1.2m BMC+ can reversibly strip 97.7% of deposited Li after resting 10 h, surpassing both ether-based and carbonate-based electrolytes in terms of performance. The longer the aging period, the more pronounced the difference becomes. The optimized 1.2m BMC+ can achieve a retention of 95.5% after one week of aging, manifesting its excellent stability and high quality of SEI.

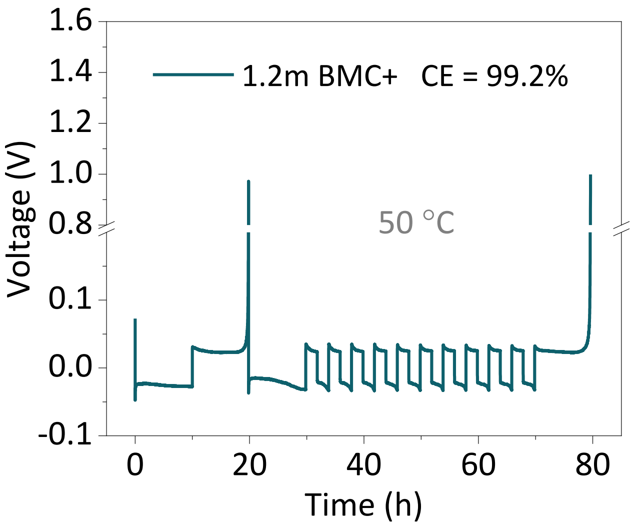

Supplementary Figure 31. Li plating/stripping CE test performed with 0.5 mA cm^{-2} and 1 mAh cm^{-2} in 1.2m BMC+ at 50 °C.

Note: It can be found that a high Li CE of 99.2% can still be achieved in 1.2m BMC+ upon elevating the temperature to 50 °C.

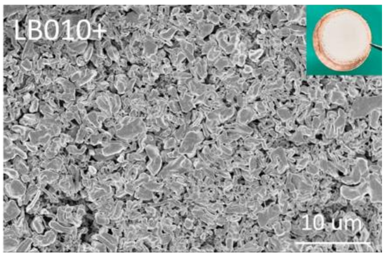

Supplementary Figure 32. SEM and optical images for the surface of Li electrodeposits after the first plating on the Cu foil at 0.5 mA cm^{-2} and 3 mAh cm^{-2} using LB010+.

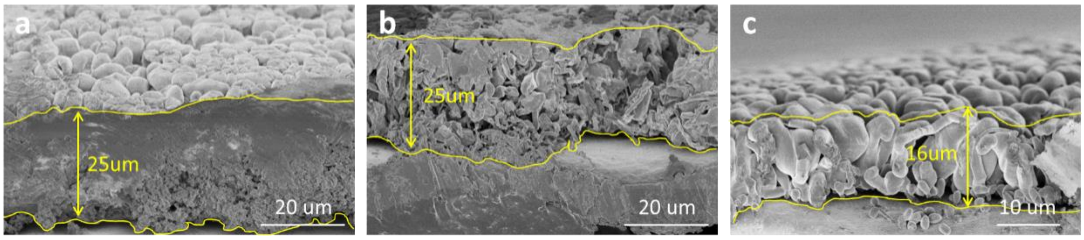

Supplementary Figure 33. SEM images for the cross-section of Li electrodeposits after the first plating on the Cu foil at 0.5 mA cm^{-2} and 3 mAh cm^{-2} using different electrolytes: (a) 1.2m DME+, (b) LB010+ and (c) 1.2m BMC+.

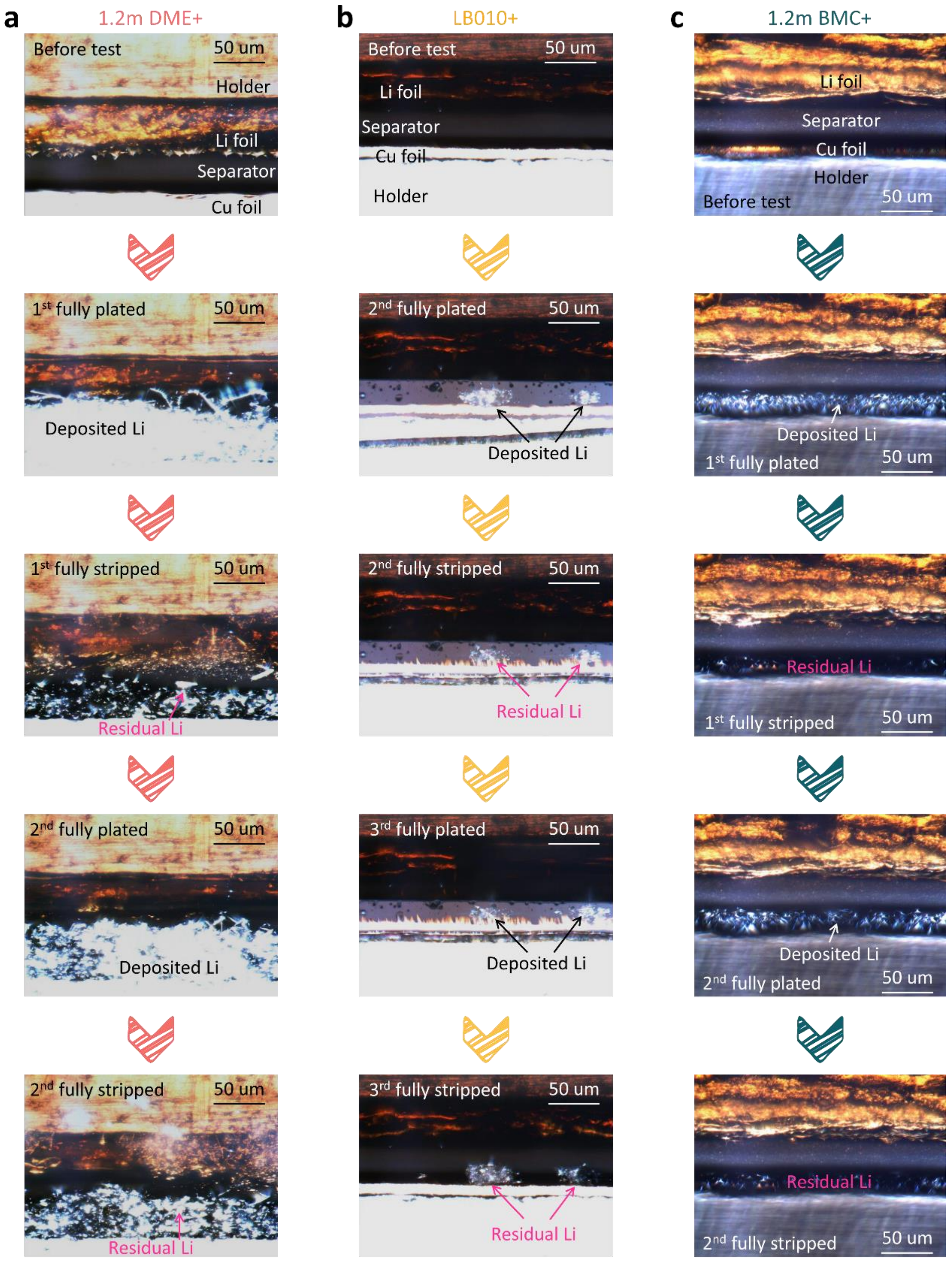

Supplementary Figure 34. In situ ECCS observations with more details and snapshots for evolution processes of Li plating/stripping in different electrolytes: (a) 1.2m DME+, (b) LB010+ and (c) 1.2m BMC+.

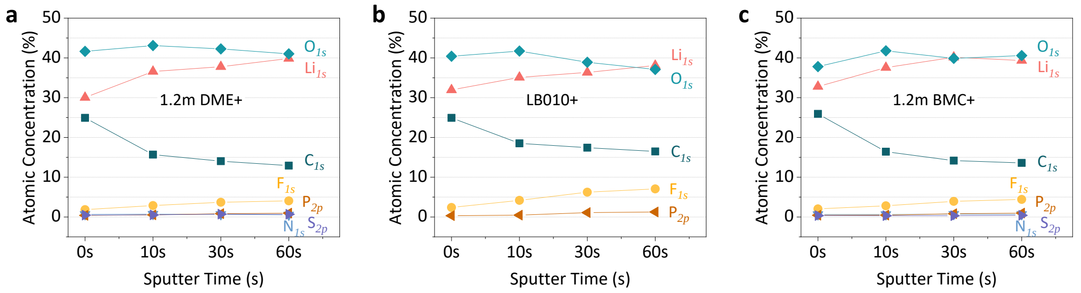

Supplementary Figure 35. XPS depth profiles of the Cu substrates with various durations of Ar^+ sputtering after one cycle of Li plating/stripping in different electrolytes: (a) 1.2m DME+, (b) LB010+ and (c) 1.2m BMC+.

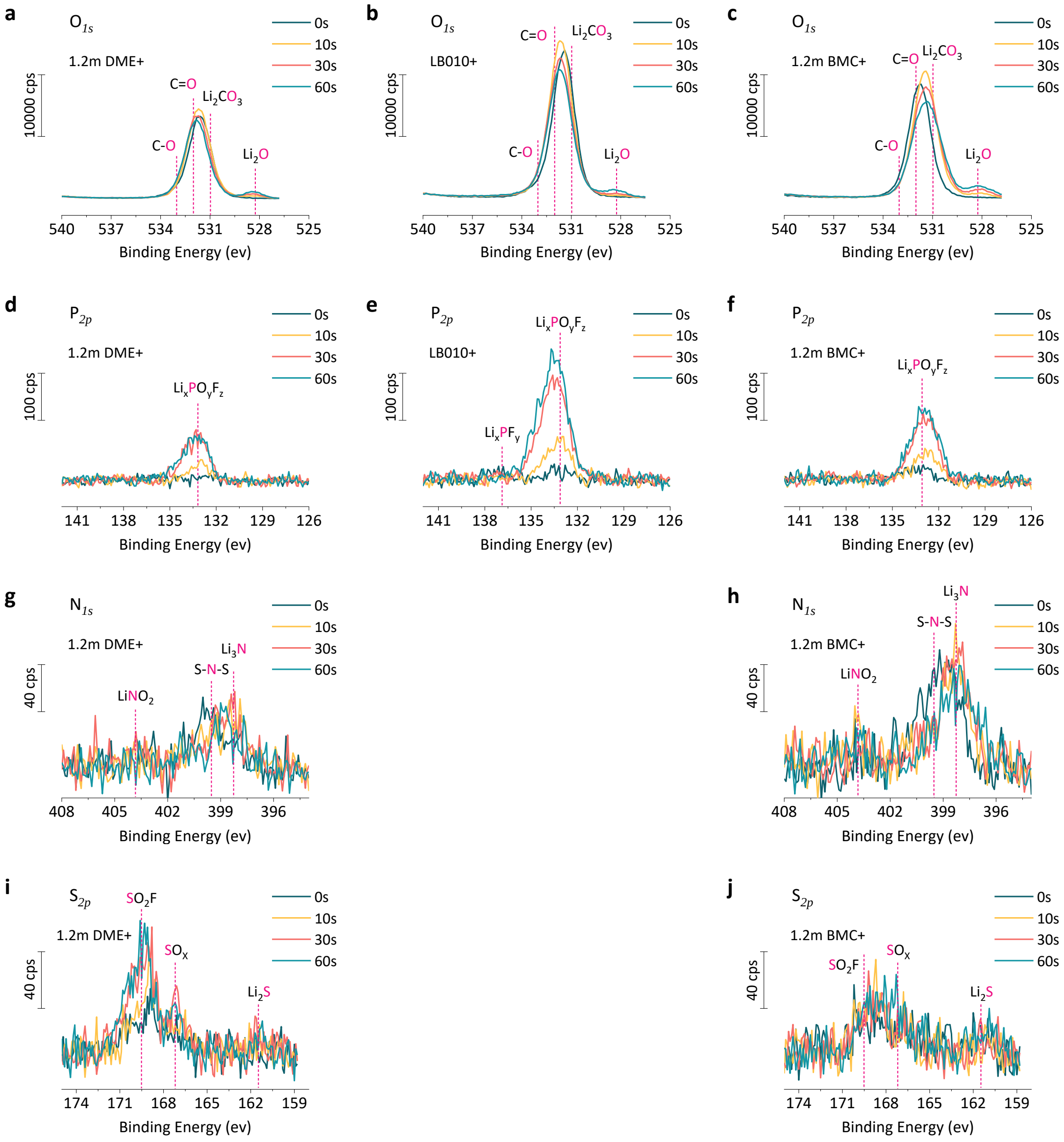

Supplementary Figure 36. XPS results of SEI formed in different additive-containing electrolytes. (a) O_{1s}, (d) P_{2p}, (g) N_{1s} and (i) S_{2p} XPS patterns of the Cu substrate with various durations of Ar^+ sputtering after one cycle of Li plating/stripping in 1.2m DME+. (b) O_{1s} and (e) P_{2p} XPS patterns of the Cu substrate with various durations of Ar^+ sputtering after one cycle of Li plating/stripping in LB010+. (c) O_{1s}, (f) P_{2p}, (h) N_{1s} and (j) S_{2p} XPS patterns of the Cu substrate with various durations of Ar^+ sputtering after one cycle of Li plating/stripping in 1.2m BMC+.

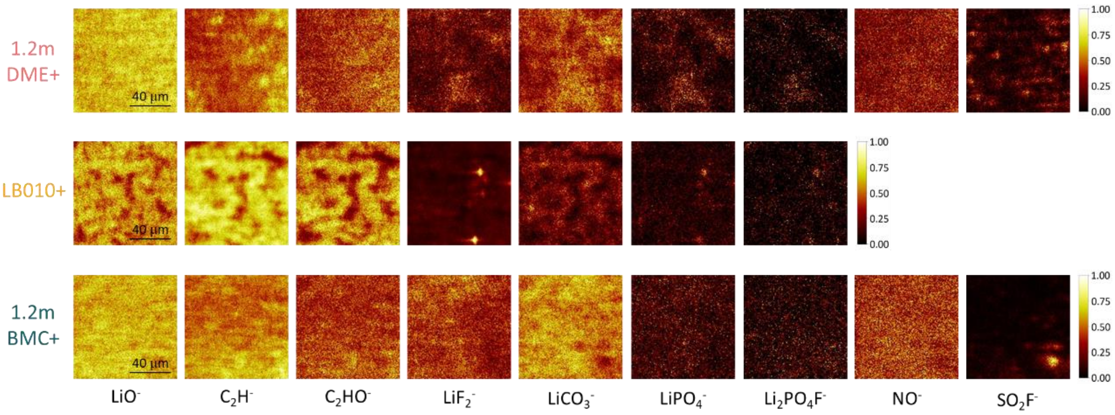

Supplementary Figure 37. Illustrative TOF-SIMS chemical mapping of the Cu substrates after one cycle of Li plating/stripping in 1.2m DME+, LB010+ and 1.2m BMC+.

Note: $LiPO_4^-$, $Li_2PO_4F^-$, NO^- fragments are found in SEIs derived by 1.2m DME+ and 1.2m BMC+, demonstrating the participation of LiDFBOP and $LiNO_3$ additives in constructing SEI. In addition, the brighter the spot, the higher the relative content. Therefore, it can be seen that the distribution of LB010+-derived SEI's constituents is extremely uneven, especially LiF_2^-, $LiPO_4^-$ and $Li_2PO_4F^-$ fragments. The SEI constituents are most evenly distributed in the sample of 1.2m BMC+.

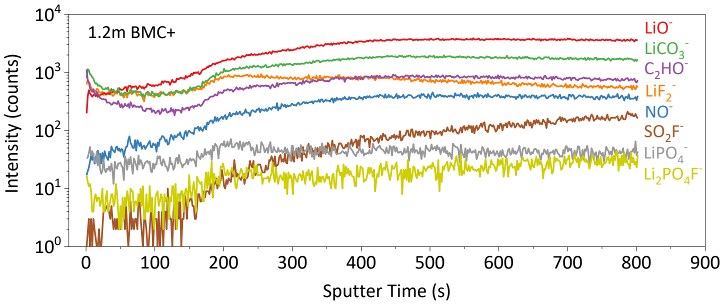

Supplementary Figure 38. TOF-SIMS depth profiles of related negatively charged fragments on the Cu substrate after one cycle of Li plating/stripping in 1.2m BMC+.

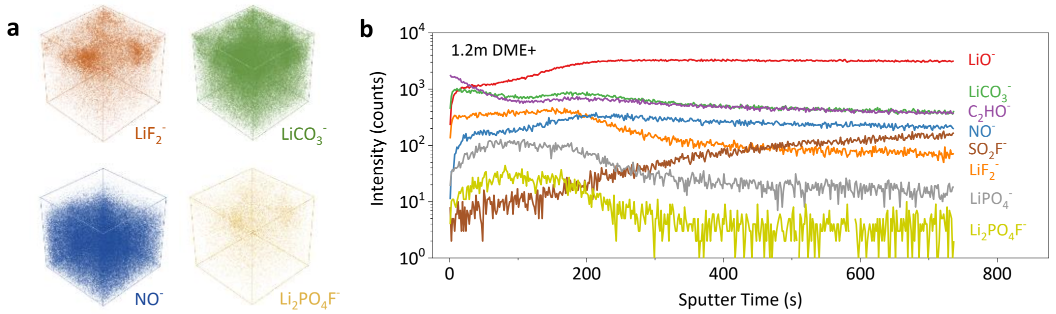

Supplementary Figure 39. TOF-SIMS analyses for SEI constructed in 1.2m DME+. (a) 3D reconstruction of the sputtered volume and (b) the depth profiles of related negatively charged fragments on the Cu substrate after one cycle of Li plating/stripping in 1.2m DME+.

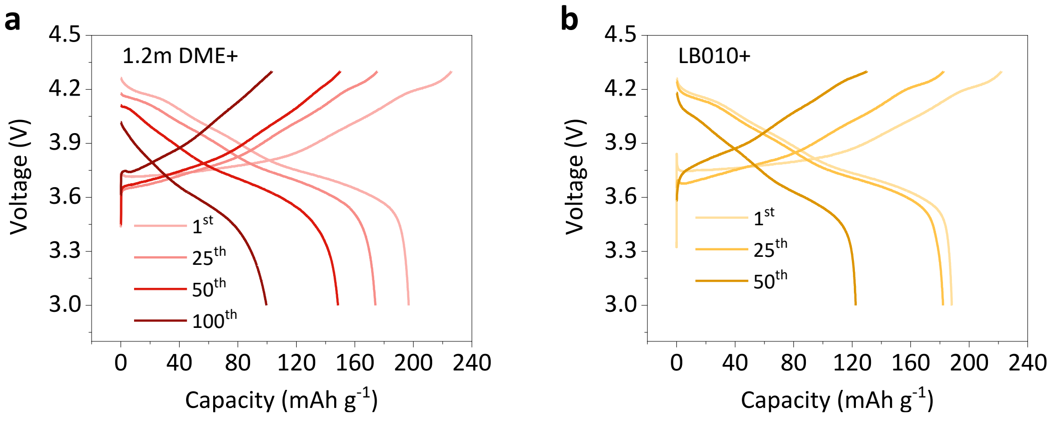

Supplementary Figure 40. Selected charge/discharge curves from 100 μm Li||4.8 mAh cm^{-2} NCM811 full cells cycled in different electrolytes: (a) 1.2m DME+ and (b) LB010+.

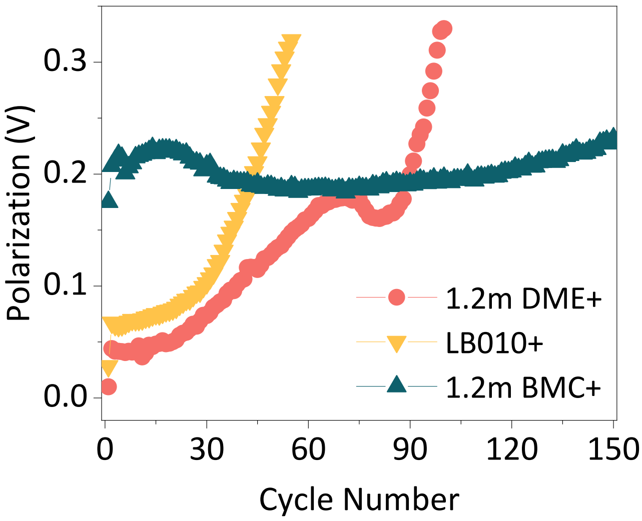

Supplementary Figure 41. Evolutions of voltage polarizations (voltage gap between charge medium voltage and discharge medium voltage) during the cycling tests on 100 μm Li||4.8 mAh cm^{-2} NCM811 full cells operated in different electrolytes.

Note: For the cells cycled in 1.2m DME+ and LB010+, voltage polarizations drastically increase upon cycling. On the contrary, there is no significant increase in voltage polarization for the cell operated in 1.2m BMC+ during cycling, indicating few side reactions.

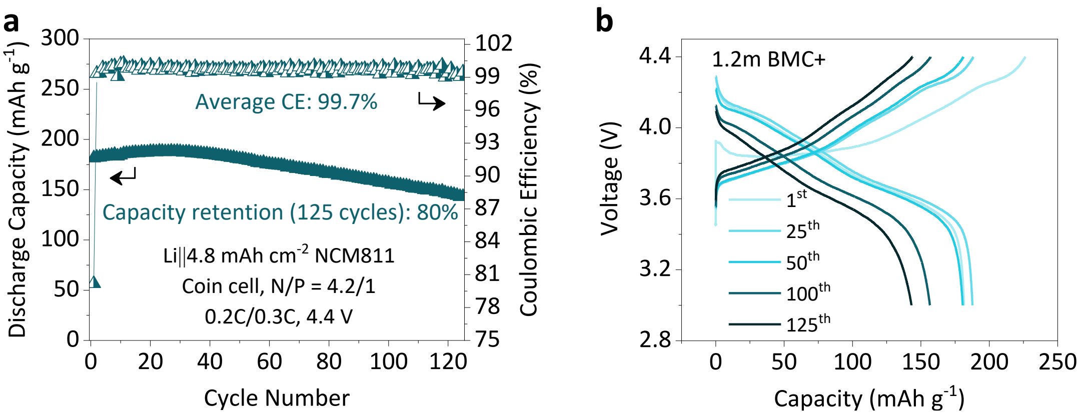

Supplementary Figure 42. Cycling performance conducted with the cut-off voltage of 4.4 V. (a) Cycling performance of 100 μm Li||4.8 mAh cm^{-2} NCM811 full cell operated at 0.2 C charge / 0.3 C discharge with lean 1.2m BMC+ (7.3 mL Ah^{-1}) in the voltage range of 3-4.4 V, and (b) corresponding selected charge/discharge curves.

Note: With the cut-off voltage of 4.4 V and a low electrolyte/cathode (E/C) ratio of 7.3 mL Ah^{-1}, the Li||NCM811 full cell operated at 0.2 C charge and 0.3 C discharge in 1.2m BMC+ presents a fairish performance with a high average CE of 99.7% that lasts for 125 cycles and achieves a capacity retention of 80%.

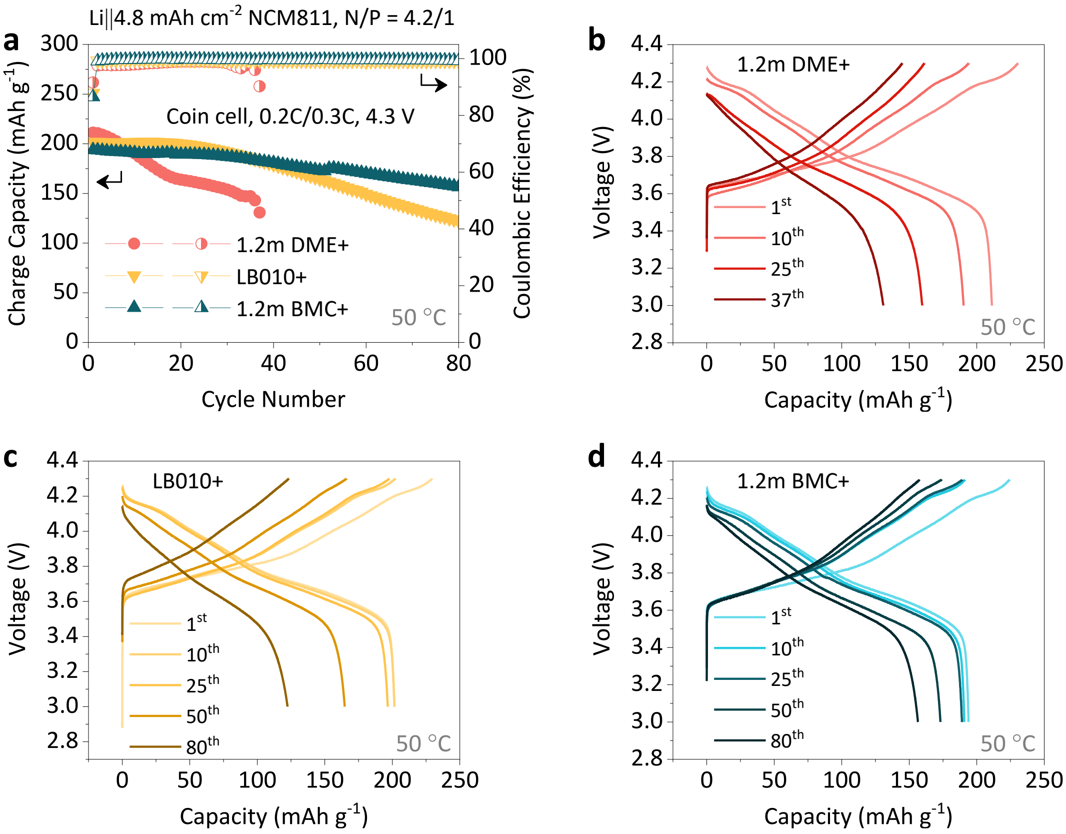

Supplementary Figure 43. Cycling performance conducted at high temperatures. (a) Cycling performance of 100 μm Li||4.8 mAh cm^{-2} NCM811 full cells operated with 0.2 C charge / 0.3 C discharge and lean electrolytes (7.3 mL Ah^{-1}) in the voltage range of 3-4.3 V at 50 °C, and corresponding selected charge/discharge curves in (b) 1.2m DME+, (c) LB010+ and (d) 1.2m BMC+.

Note: In terms of high-temperature cycling for the Li||NCM811 full cell, it maintains 81% of its initial capacity after undergoing 80 cycles with an average CE of 99.6% in 1.2m BMC+, surpassing its counterparts using 1.2m DME+ (37 cycles with a capacity retention of 62% and an average CE of 98.0%) and LB010+ (80 cycles with a capacity retention of 60% and an average CE of 99.3%).

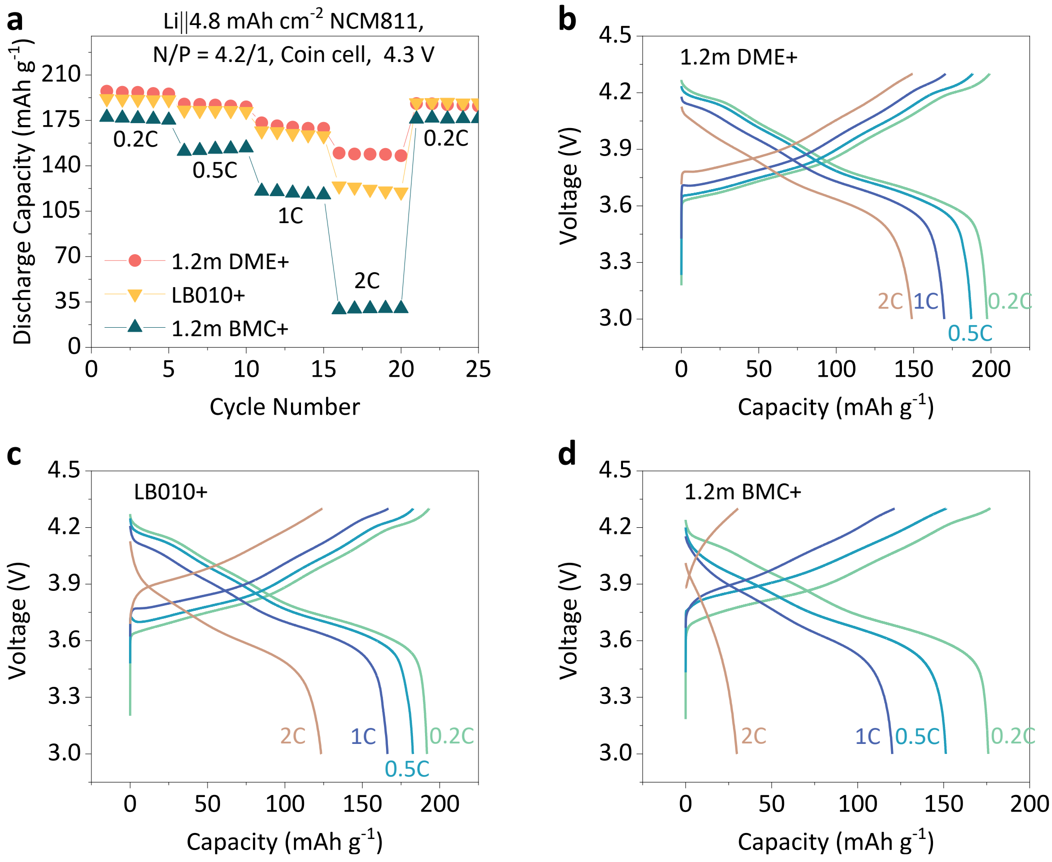

Supplementary Figure 44. Rate performance of Li||NCM811 full cells. (a) Rate capability of 100 μm Li||NCM811 full cells operated in various lean electrolytes (7.3 mL Ah^{-1}) in the voltage range of 3-4.3 V, and corresponding selected charge/discharge curves in (b) 1.2m DME+, (c) LB010+ and (d) 1.2m BMC+.

Note: Supplementary Fig. 44 presents the rate performance of the Li||NCM811 full cell operated in 1.2m BMC+, compared with those operated in 1.2m DME+ and LB010+ counterparts. While increasing the charge/discharge rate from 0.2 C to 0.5 C, 1 C and 2 C, the full cell with 1.2m DME+ delivers discharge capacities of 187 mAh g^{-1}, 173 mAh g^{-1} and 150 mAh g^{-1} respectively (capacity retention: 95%, 87% and 76%). Similarly, the full cell with LB010+ demonstrates discharge capacities of 183 mAh g^{-1}, 166 mAh g^{-1} and 124 mAh g^{-1} (capacity retention: 95%, 86% and 64%), while the full cell with 1.2m BMC+ displays discharge capacities of 152 mAh g^{-1}, 121 mAh g^{-1} and 30 mAh g^{-1} (capacity retention: 86%, 69% and 17%). It can be obviously observed that the low ionic conductivity of the BMC-based electrolyte, resulting from the high viscosity of BMC, impedes the rate performance of the Li||NCM811 full cell. In practical applications, it is feasible to incorporate low-viscosity co-solvents or diluents into the BMC-based electrolyte to enhance its conductivity and achieve improvements in the rate performance of batteries while preserving the function of BMC in terms of cycling and safety performance.

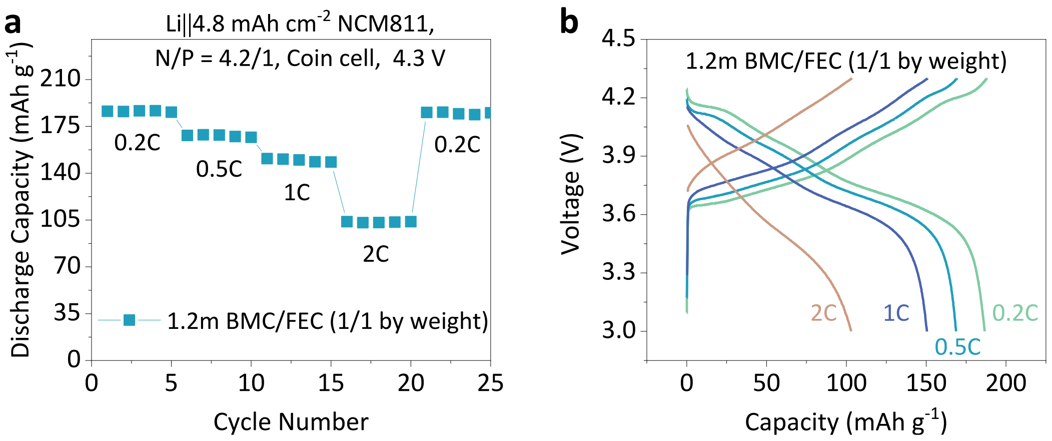

Supplementary Figure 45. Rate performance of Li||NCM811 full cells. (a) Rate capability of 100 μm Li||4.8 mAh cm^{-2} NCM811 full cell operated in lean 1.2m BMC/FEC (1/1 by weight) (7.3 mL Ah^{-1}) with the voltage range of 3-4.3 V, and (b) corresponding selected charge/discharge curves.

Note: Upon the introduction of FEC as a co-solvent, the 1.2m BMC/FEC (1/1 by weight, without additives) successfully enables 100 μm Li||4.8 mAh cm^{-2} NCM811 full cell to provide a capacity of more than 100 mAh g^{-1} at 2 C, which is superior to 1.2m BMC+.

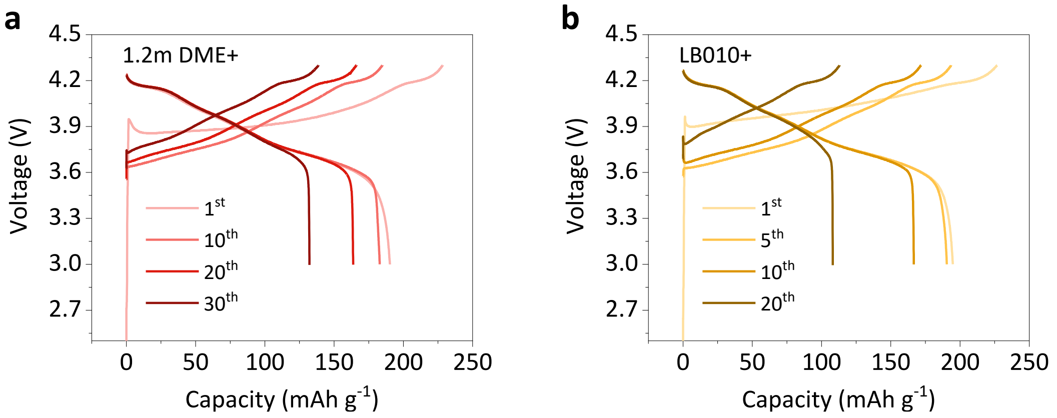

Supplementary Figure 46. Selected charge/discharge curves from anode-free Cu||2.0 mAh cm^{-2} NCM811 coin cells cycled in different electrolytes: (a) 1.2m DME+ and (b) LB010+.

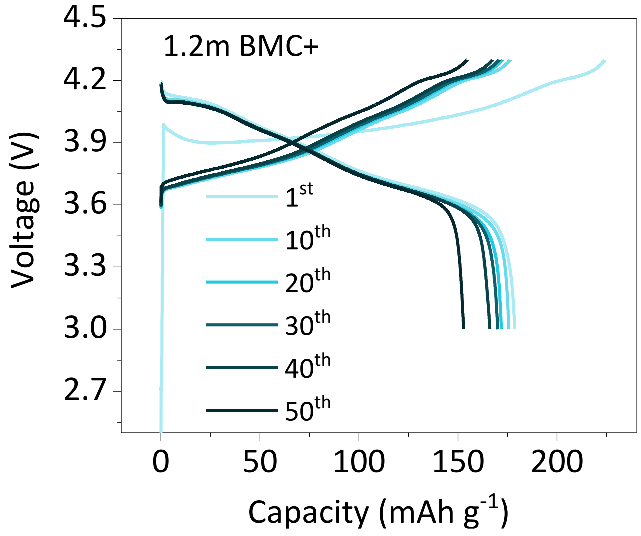

Supplementary Figure 47. Selected charge/discharge curves from anode-free Cu||2.0 mAh cm^{-2} NCM811 coin cells cycled in 1.2m BMC+.

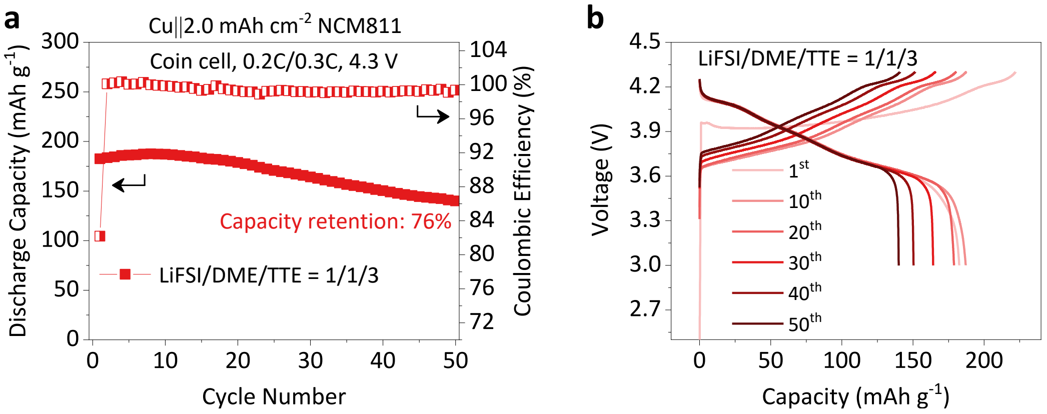

Supplementary Figure 48. Cycling performance of anode-free Cu||NCM811 coin cells. (a) Cycling performance of anode-free Cu||2.0 mAh cm^{-2} NCM811 coin cells operated at 0.2 C charge / 0.3 C discharge with lean electrolyte (5 mL Ah^{-1}) in the voltage range of 3-4.3 V, and (b) corresponding selected charge/discharge curves in the critically acclaimed LHCE (LiFSI/DME/1,1,2,2-tetrafluoroethyl-2,2,3,3-tetrafluoropropyl ether (TTE) = 1/1/3 by mol).

Note: Based on the critically acclaimed LHCE (LiFSI/DME/TTE = 1/1/3 by mol)[10-12], the anode-free Cu||2.0 mAh cm^{-2} NCM811 coin cell exhibits a capacity retention of 76% after 50 cycles, inferior to that operated in our 1.2m BMC+ (86%).

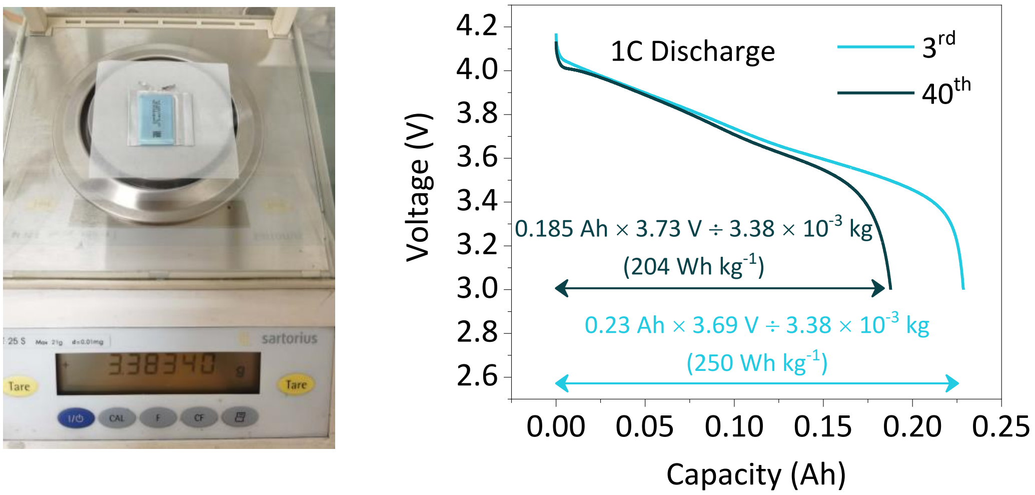

Supplementary Figure 49. The total weight of industrial Cu||3.2 mAh cm^{-2} NCM811 pouch cell operated in 1.2m BMC+, and the calculated energy density of the pouch cell (1 C discharge) after 3 and 40 cycles.

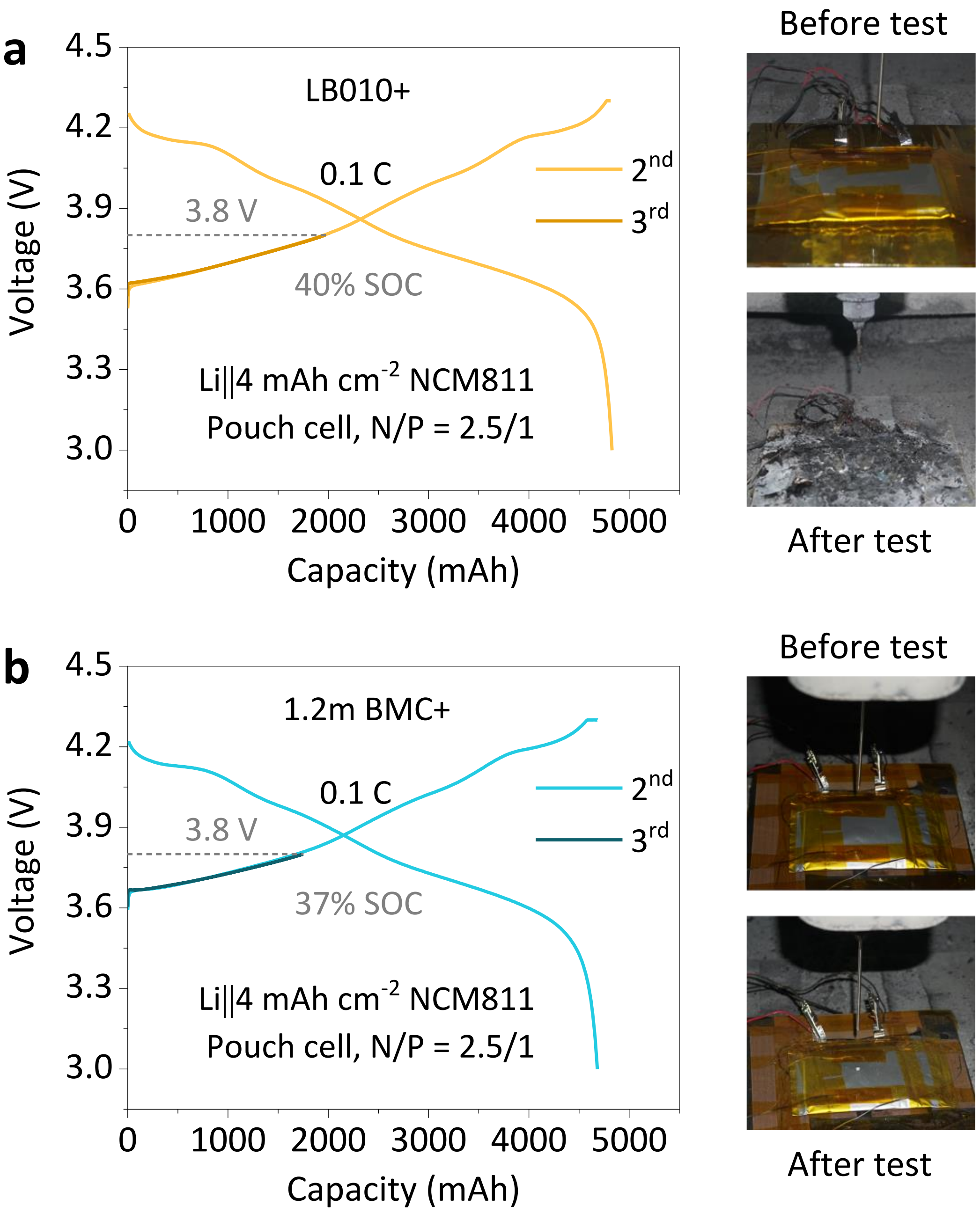

Supplementary Figure 50. Nail penetration tests of Ah-level Li||NCM811 pouch cells. Charge/discharge curves of 5 Ah Li||NCM811 (4 mAh cm^{-2}, N/P = 2.5/1, Electrolyte/Cathode = 2 g Ah^{-1}) pouch cells operated in (a) LB010+ and (b) 1.2m BMC+ at a rate of 0.1 C, and corresponding photos before and after nail penetration tests (The nail penetration tests are conducted on the pouch cells after they have been charged to the voltage of 3.8 V).

Note: The nail penetration tests were conducted on Ah-level pouch cells to explore the safety performance. 5 Ah Li||NCM811 pouch cells (4 mAh cm^{-2}, N/P = 2.5/1, Electrolyte/Cathode = 2 g Ah^{-1}) are prepared with LB010+ and 1.2m BMC+ electrolytes, respectively. After two formation cycles, both cells were charged to a voltage of 3.8 V (equivalent to approximately 40% SOC) for nail penetration tests. A steel nail with a diameter of 3 mm was moving at a speed of 25 mm s^{-1} to vertically penetrate through the cell. As depicted in Supplementary Fig. 50, the cell with LB010+ electrolyte unfortunately

failed in the nail penetration and it triggered thermal runaway resulting in violent explosive combustion (Supplementary Fig. 50a and Supplementary Movie 10). As a comparison, the cell with 1.2m BMC+ electrolyte successfully passed the nail penetration test (Supplementary Fig. 50b and Supplementary Movie 9), which can be inferred with the lower heat release, exceptional thermal stability and non-flammability characteristics of BMC. The results well indicated that BMC solvent exhibited better safety performance.

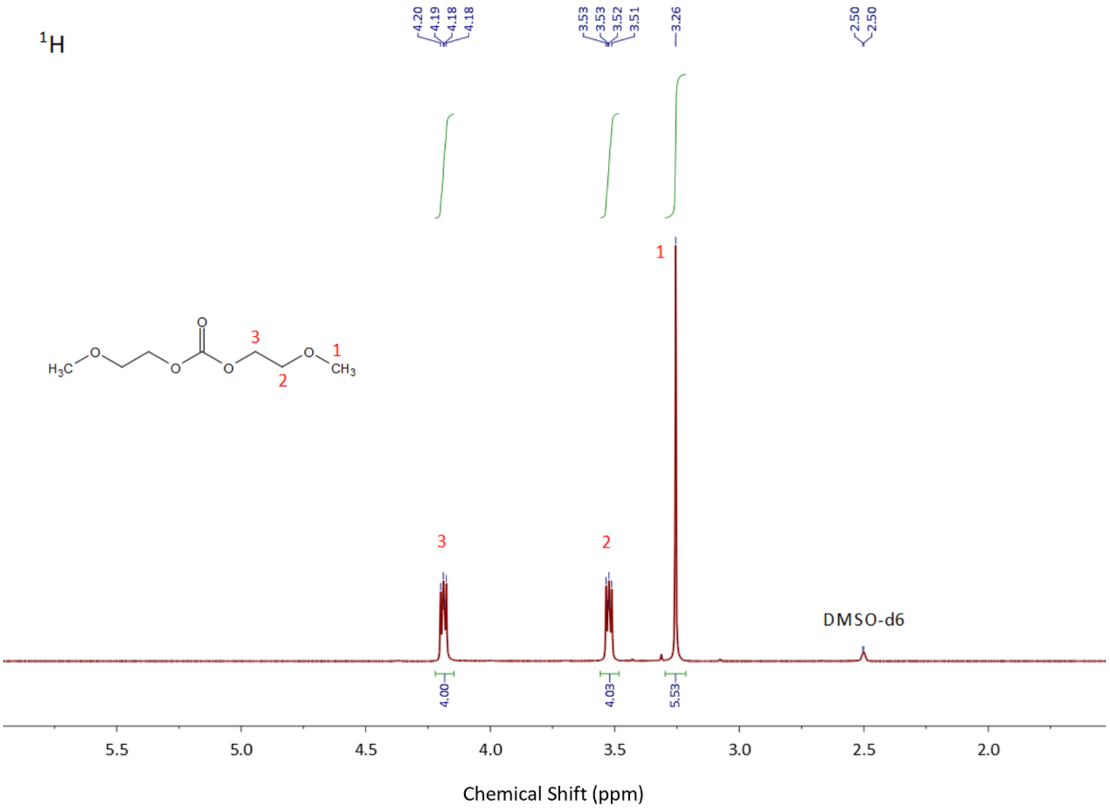

Supplementary Figure 51. ^{1}H NMR of synthesized BMC. ^{1}H NMR (400 MHz, DMSO-d6) δ 4.19 (dd, J = 5.4, 3.7 Hz, 4H), 3.52 (dd, J = 5.4, 3.7 Hz, 4H), 3.26 (s, 6H).

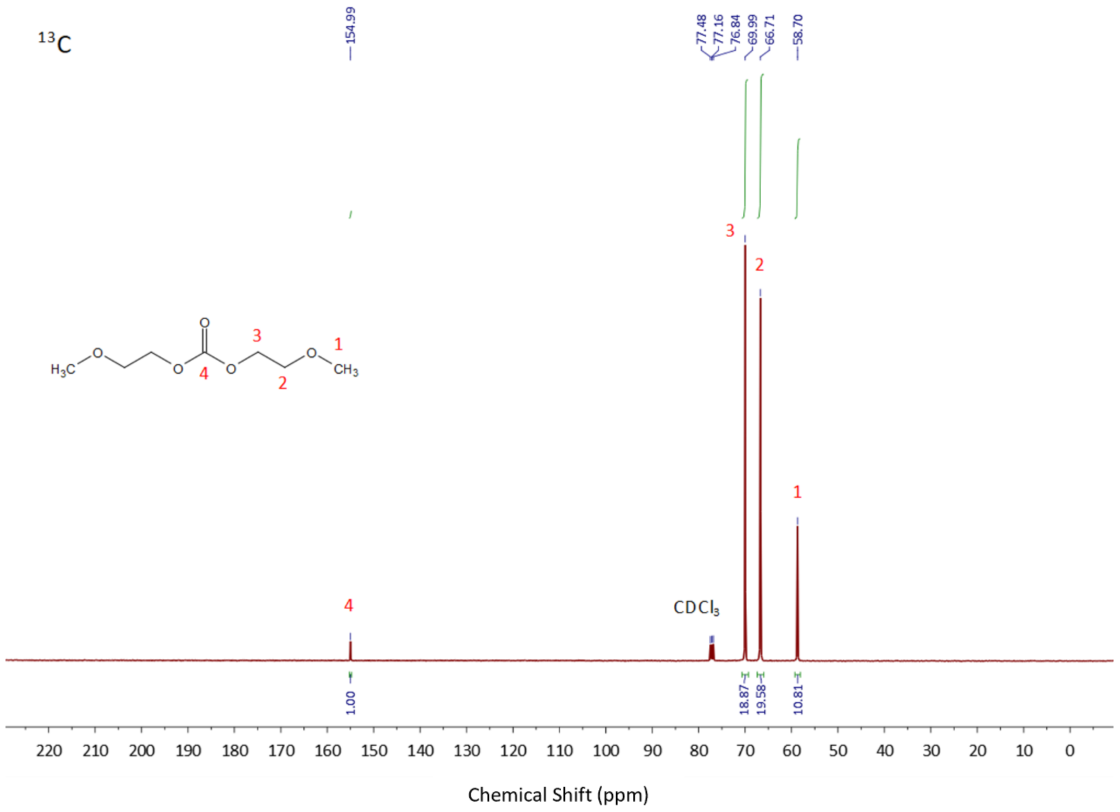

Supplementary Figure 52. ^{13}C NMR of synthesized BMC. ^{13}C NMR (100 MHz, $CDCl_3$) δ 154.99 (s), 69.99 (s), 66.71 (s), 58.70 (s).

Supplementary Table 1. Costs of precursors for the synthesis of BMC.

Precursors	Specifications & Purity	Costs*
Dimethyl carbonate	anhydrous, ≥99%	$212 / 2 L
2-Methoxyethanol	anhydrous, 99.8%	$458 / 2 L

* The costs values are obtained from Sigma-Aldrich. The prices can be further decreased if large-scale products are directly purchased from chemical factories.

Supplementary Table 2. Statistical results of various Li^+-BMC coordination configurations. The results are based on all Li^+ primary solvation structures summarized from the MD simulation of 1.2m BMC. (Li^+-BMC_1, Li^+-BMC_2, Li^+-BMC_3, Li^+-BMC_4 and Li^+-BMC_5 can correspond to Li^+-BMC complex-1, Li^+-BMC complex-2, Li^+-BMC complex-3, Li^+-BMC complex-4 and Li^+-BMC complex-5 shown in DFT results, respectively.)

Coordination configurations of Li^+-BMC	Counts	Proportions (%)
Li^+-BMC_1 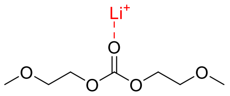	62	70.4
Li^+-BMC_2 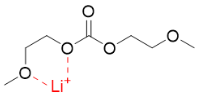	9	10.2
Li^+-BMC_3 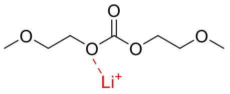	5	5.7
Li^+-BMC_4 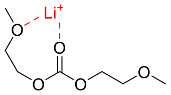	7	8.0
Li^+-BMC_5 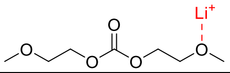	5	5.7

Note: After summarizing all Li^+ primary solvation structures in MD simulations, followed by meticulously counting the number of various Li^+-BMC coordination configurations, their respective proportions are shown in Supplementary Table 2. The dominant solvation configuration is represented by Li^+-BMC_1 (coordinated to Li^+ via one carbonyl O atom), constituting a remarkable 70.4% of all solvated BMC molecules. Subsequently, the chelation configurations, Li^+-BMC_2 (coordinated to Li^+ via one single bond O atom in the carbonate group and one ether O atom) and Li^+-BMC_4 (coordinated to Li^+ via one carbonyl O atom and one ether O atom) account for 10.2% and 8%, respectively. As for Li^+-BMC_3 (coordinated to Li^+ via one single bond O atom in the carbonate group) and Li^+-BMC_5 (coordinated to Li^+ via one ether O atom), they contribute equally with a proportion of 5.7%. Li^+-BMC_1 representing the dominant coordination configuration is in line with the Raman results that indicate the carbonyl oxygen serves as the primary solvation site for BMC, while the relatively low content of Li^+-BMC_2 and Li^+-BMC_4 with strong chelation configurations is consistent with the limited solubility of $LiNO_3$ in BMC and only slight blue shift of ether C-O-C stretching vibrations observed in Raman spectra after the introduction of LiFSI in BMC.

Supplementary Table 3. Fitting details for the Raman spectra of 1.2m DME, 1.2m DMC and 1.2m BMC in 680-780 cm^{-1} (S-N-S bending vibration of FSI^-).

	**Free FSI^-**			**CIP**			**AGG**		
	**Peak center**	**FWHM**	**Area (proportion)**	**Peak center**	**FWHM**	**Area (proportion)**	**Peak center**	**FWHM**	**Area (proportion)**
**1.2m DME**	719.0 (cm^{-1})	18.1 (cm^{-1})	35675.0 (65.3%)	730.6 (cm^{-1})	19.2 (cm^{-1})	13161.9 (24.1%)	742.3 (cm^{-1})	27.8 (cm^{-1})	5803.2 (10.6%)
**1.2m DMC**	719.0 (cm^{-1})	18.1 (cm^{-1})	10437.6 (42.7%)	730.6 (cm^{-1})	19.2 (cm^{-1})	10517.9 (43.0%)	742.3 (cm^{-1})	27.8 (cm^{-1})	3490.6 (14.3%)
**1.2m BMC**	719.0 (cm^{-1})	18.1 (cm^{-1})	21212.9 (38.2%)	730.6 (cm^{-1})	19.2 (cm^{-1})	22886.8 (41.2%)	742.3 (cm^{-1})	27.8 (cm^{-1})	11472.6 (20.6%)

Supplementary Table 4. Comparisons of average Li plating/stripping CEs obtained in our optimized electrolyte system and those in previously reported ether-based and carbonate-based electrolytes that are in the form of normal concentration electrolytes, HCEs and LHCEs.

Electrolyte formula	Current; Capacity	CE/Testing cycles	Ref.
**1.2m LiFSI in BMC + 0.75 wt.% $LiNO_3$ + 1 wt.% LiDFBOP**	**0.5 mA cm^{-2}; 1 mAh cm^{-2}**	**99.4%/10 cycles**<br>**99.3%/125 cycles**	**This work**
1M $LiPF_6$ in EC/DEC (1:1 by vol.) + 0.2 wt.%CuF_2 + 1 wt.% $LiNO_3$	0.5 mA cm^{-2}; 0.5 mAh cm^{-2}	98.1%/10 cycles	[13]
1M $LiPF_6$ in FEC/BTC (3:7 by vol.)	0.5 mA cm^{-2}; 1 mAh cm^{-2}	98.8%//between 210-300 cycle	[14]
1M $LiPF_6$ in EC/DEC (1:1 by vol.) + 15 wt.% FEC + 1 wt.% LiDFOB	0.5 mA cm^{-2}; 1 mAh cm^{-2}	99%/10 cycles	[15]
1M LiFSI in BME	0.5 mA cm^{-2}; 0.5 mAh cm^{-2}	98.5%/10 cycles	[16]
1M LiFSI in cFTOF	0.5 mA cm^{-2}; 1 mAh cm^{-2}	98.6%/300 cycles	[17]
2M LiFSI in DMP	0.5 mA cm^{-2}; 1 mAh cm^{-2}	99.2%/10 cycles	[18]
1.8M LiFSI in DPE	0.5 mA cm^{-2}; 1 mAh cm^{-2}	99.42%/10 cycles	[19]
2M LiFSI in TFDMP	0.5 mA cm^{-2}; 1 mAh cm^{-2}	99.6%/10 cycles	[20]
2M LiFSI in BFE	0.5 mA cm^{-2}; 1 mAh cm^{-2}	99.75%/10 cycles	[21]
7m LiFSI in FEC	0.25 mA cm^{-2}; 0.5 mAh cm^{-2}	98.75%/400 cycles	[22]

10M LiFSI in DMC	0.2 mA cm^{-2}; 1 mAh cm^{-2}	99.2%/between 160-200 cycle	[23]
10M LiFSI in EC/DMC	0.2 mA cm^{-2}; 1 mAh cm^{-2}	99.3%/between 100-250 cycle	[23]
12M LiFSI in DME	1 mA cm^{-2}; 1 mAh cm^{-2}	99.2%/250 cycles	[24]
4M LiFSI in DEE	0.5 mA cm^{-2}; 1 mAh cm^{-2}	99.38%/10 cycles	[7]
LiFSI-DMC-TTE (1:1.5:1.5 by mol)	1 mA cm^{-2}; 1 mAh cm^{-2}	98.6%/400 cycles	[25]
1.2M LiFSI in DMC/BTFE (1:2 by mol)	0.5 mA cm^{-2}; 1 mAh cm^{-2}	99.3%/10 cycles	[26]
LiFSI-ClDEE-TTE (1:1.6:3 by mol)	0.5 mA cm^{-2}; 1 mAh cm^{-2}	99.2%/10 cycles	[27]
1M LiFSI in DME/BTFE (1:5 by vol.)	0.5 mA cm^{-2}; 1 mAh cm^{-2}	99.4%/20 cycles	[28]
LiFSI-FDEE-TTE (1:1.65:3 by mol)	0.5 mA cm^{-2}; 1 mAh cm^{-2}	99.4%/10 cycles	[29]
1M LiFSI in DME/TFEO (1:9 by wt.)	0.5 mA cm^{-2}; 1 mAh cm^{-2}	99.5%/10 cycles	[30]

Supplementary References

1 Yao, N. et al. An atomic insight into the chemical origin and variation of the dielectric constant in liquid electrolytes. *Angew. Chem. Int. Ed.* **60**, 21473–21478 (2021).

2. Fang W. et al. Constructing inorganic-rich solid electrolyte interphase via abundant anionic solvation

sheath in commercial carbonate electrolytes. *Nano Energy* **104**, 107881 (2022).

3. Shi, Q., Zhong, Y., Wu, M. & Wang, H. High-capacity rechargeable batteries based on deeply cyclable lithium metal anodes. *Proc. Natl. Acad. Sci. USA* **115**, 5676−5680 (2018).

4. Yan C. et al. Lithium nitrate solvation chemistry in carbonate electrolyte sustains high-voltage lithium metal batteries. *Angew. Chem. Int. Ed.* **57**, 14055−14059 (2018).

5. Choo, Y. et al. Complete electrochemical characterization and limiting current of polyacetal electrolytes. *J. Electrochem. Soc.* **169**, 020538 (2022).

6. Kerner, M., Plylahan, N., Scheers, J. & Johansson P. Thermal stability and decomposition of lithium bis (fluorosulfonyl) imide (LiFSI) salts. *RSC Adv.* **6**, 23327−23334 (2016).

7. Chen, Y. et al. Steric effect tuned ion solvation enabling stable cycling of high-voltage lithium metal battery. *J. Am. Chem. Soc.* **143**, 18703−18713 (2021).

8. Zhuang, J. et al. A self-healing interface on lithium metal with lithium difluoro (bisoxalato) phosphate for enhanced lithium electrochemistry. *J. Mater. Chem. A* **7**, 26002−26010 (2019).

9. Yan, C. et al. Regulating the inner Helmholtz plane for stable solid electrolyte interphase on lithium metal anodes. *J. Am. Chem. Soc.* **141**, 9422−9429 (2019).

10. Ren, X. et al. Enabling high-voltage lithium-metal batteries under practical conditions. *Joule* **3**, 1662–1676 (2019).

11. Niu, C. et al. Balancing interfacial reactions to achieve long cycle life in high-energy lithium metal batteries. *Nat. Energy* **6**, 723–732 (2021).

12. Xu, R. et al. Designing and demystifying the lithium metal interface toward highly reversible batteries. *Adv. Mater.* **33**, 2105962 (2021).

13. Zhang, X.-Q. et al. Highly stable lithium metal batteries enabled by regulating the solvation of

lithium ions in nonaqueous electrolytes. *Angew. Chem. Int. Ed.* **57**, 5301–5305 (2018).

14. Xiao, P. et al. A nonflammable electrolyte for ultrahigh-voltage (4.8 V-class) Li||NCM811 cells with a wide temperature range of 100 °C. *Energy Environ. Sci.* **15**, 2435–2444 (2022).

15. Li, G.-X. et al. A superior carbonate electrolyte for stable cycling Li metal batteries using high Ni cathode. *ACS Energy Lett.* **7**, 2282–2288 (2022).

16. Wu, J. et al. Unique tridentate coordination tailored solvation sheath towards highly stable lithium metal batteries. *Adv. Mater.* **35**, 2303347 (2023).

17. Zhou, T., Zhao, Y., Kazzi, M. E., Choi, J. W. & Coskun, A. Integrated ring-chain design of a new fluorinated ether solvent for high-voltage lithium-metal batteries. *Angew. Chem. Int. Ed.* **61**, e202115884 (2022).

18. Park, E. et al. Exploiting the steric effect and low dielectric constant of 1,2-dimethoxypropane for 4.3 V lithium metal batteries. *ACS Energy Lett.* **8**, 179−188 (2023).

19. Li, Z. et al. Non-polar ether-based electrolyte solutions for stable high-voltage non-aqueous lithium metal batteries. *Nat. Commun.* **14**, 868 (2023).

20. Zhao, Y., Zhou, T., Mensi, M., Choi, J. W. & Coskun, A. Electrolyte engineering via ether solvent fluorination for developing stable non-aqueous lithium metal batteries. *Nat. Commun.* **14**, 299 (2023).

21. Zhang, G. et al. A monofluoride ether-based electrolyte solution for fast-charging and low-temperature non-aqueous lithium metal batteries. *Nat. Commun.* **14**, 1081 (2023).

22. Suo, L. et al. Fluorine-donating electrolytes enable highly reversible 5-V-class Li metal batteries. *Proc. Natl Acad. Sci. USA* **115**, 1156–1161 (2018).

23. Fan, X. et al. Highly fluorinated interphases enable high-voltage Li-metal batteries. *Chem* **4**, 174–185 (2018).

24. Zheng, J. et al. Manipulating electrolyte and solid electrolyte interphase to enable safe and efficient Li–S batteries. *Nano Energy* **50**, 431–440 (2018).

25. Piao, N. et al. Countersolvent electrolytes for lithium-metal batteries. *Adv. Energy Mater.* **10**, 1903568 (2020).

26. Chen, S. et al. High-voltage lithium-metal batteries enabled by localized high-concentration electrolytes. *Adv. Mater.* **30**, e1706102 (2018).

27. Tan, L. et al. Intrinsic nonflammable ether electrolytes for ultrahigh-voltage lithium metal batteries enabled by chlorine functionality. *Angew. Chem. Int. Ed.* **61**, e202203693 (2022).

28. Holoubek, J. et al. Electrolyte design implications of ion-pairing in low-temperature Li metal batteries. *Energy Environ. Sci.* **15**, 1647–1658 (2022).

29. Ruan, D. et al. Solvent versus anion chemistry: unveiling the structure-dependent reactivity in tailoring electrochemical interphases for lithium-metal batteries. *JACS Au* **3**, 953–963 (2023).

30. Cao, X. et al. Monolithic solid-electrolyte interphases formed in fluorinated orthoformate-based electrolytes minimize Li depletion and pulverization. *Nat. Energy* **4**, 796–805 (2019).
