# Supplementary material for: Hybridizing carbonate and ether at molecular scales for high-energy and high-safety lithium metal batteries
Source: Nat Commun. 2024 Apr 15;15:3217. doi: 10.1038/s41467-024-47448-5 (PMC11018806; doi:10.1038/s41467-024-47448-5)
Supplement: Supplementary file 3 — Description of Additional Supplementary Information [file 41467_2024_47448_MOESM3_ESM.pdf]

Description of Additional Supplementary Information

Supplementary Movie 1: The flammability test for 1.2m DME.

Supplementary Movie 2: The flammability test for 1.2m DMC.

Supplementary Movie 3: The flammability test for 1.2m DME/DMC.

Supplementary Movie 4: The flammability test for LB010.

Supplementary Movie 5: The flammability test for 1.2m BMC.

Supplementary Movie 6: In situ ECCS observations for evolution processes of Li plating/stripping in 1.2m DME+.

Supplementary Movie 7: In situ ECCS observations for evolution processes of Li plating/stripping in LB010+.

Supplementary Movie 8: In situ ECCS observations for evolution processes of Li plating/stripping in 1.2m BMC+.

Supplementary Movie 9: The nail penetration test for the pouch cell operated with 1.2m BMC+.

Supplementary Movie 10: The nail penetration test for the pouch cell operated with LB010+.
